# Ca^2+^‐ and cGAMP‐Contained Semiconducting Polymer Nanomessengers for Radiodynamic‐Activated Calcium Overload and Immunotherapy

**DOI:** 10.1002/advs.202411739

**Published:** 2024-12-16

**Authors:** Danling Cheng, Libai Luo, Qin Zhang, Zheming Song, Yiduo Zhan, Wenzhi Tu, Jingchao Li, Qiming Ma, Xianchang Zeng

**Affiliations:** ^1^ Institute of Immunology Zhejiang University School of Medicine Hangzhou 310009 China; ^2^ Oncology Chemotherapy Department Affiliated Hospital of Youjiang Medical University for Nationalities and Key Laboratory of Research on Clinical Molecular Diagnosis for High Incidence Diseases in Western Guangxi of Guangxi Higher Education Institutions Baise 533000 China; ^3^ Institute of Translational Medicine Shanghai University Shanghai 200444 China; ^4^ State Key Laboratory for Modification of Chemical Fibers and Polymer Materials College of Biological Science and Medical Engineering Donghua University Shanghai 201620 China; ^5^ Department of Radiation Oncology Shanghai General Hospital Shanghai Jiao Tong University School of Medicine Shanghai 201620 China; ^6^ Department of General Surgery The First Affiliated Hospital of Gannan Medical University Ganzhou 341000 China

**Keywords:** calcium overload, immunotherapy, radiodynamic therapy, second messengers, smart nanotherapeutic

## Abstract

Various second messengers exert some vital actions in biological systems, including cancer therapy, but the therapeutic efficacy is often need to be improved. A semiconducting polymer nanomessenger (TCa/SPN/a) consisting of two second messengers, calcium ion (Ca^2+^) and cyclic guanosine monophosphate‐adenosine monophosphate (cGAMP) for metastatic breast cancer therapy, is reported here. Such a TCa/SPN/a is constructed to exhibit X‐ray response for the activatable delivery of mitochondria‐targeting Ca compound and cGAMP as stimulator of interferon genes (STING) agonist. With X‐ray irradiation, TCa/SPN/a could generate singlet oxygen (^1^O_2_) via radiodynamic effect for ablating solid tumors and improving the tumor immunogenicity by inducing immunogenic cell death (ICD). Furthermore, the released mitochondria‐targeting Ca compounds show a high binging effect on mitochondria and cause reactive oxygen species (ROS) generation and mitochondria damage via calcium overload, while cGAMP boosts immunological effect through activating STING pathway. In this way, TCa/SPN/a enables a radiodynamic‐activated calcium overload and immunotherapy to obviously inhibit the growths of bilateral tumors and also abolish tumor metastasis in metastatic breast cancer mouse models. This article should demonstrate the first smart dual‐functional nanotherapeutic containing two second messengers for precise and specific cancer therapy.

## Graphical abstract

A Ca^2+^‐ and cyclic guanosine monophosphate‐adenosine monophosphate (cGAMP)‐contained nanomessenger is reported for breast cancer therapy. The nanomessenger exhibits X‐ray response for activatable delivery of mitochondria‐targeting Ca compound and cGAMP, leading to mitochondria damage via calcium overload, immunogenic cell death induction, and immunological effect activation. This therapeutic strategy inhibits bilateral tumor growth and abolishes tumor metastasis in breast cancer mouse models.

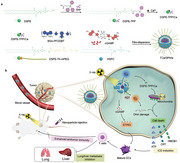

## 1 Introduction

Second messengers are a type of signaling molecules that can regulate multifarious physiological actions including cell proliferation, differentiation, migration, maturation, and apoptosis.^[^ ^1^ ^]^ Recently, second messengers have also been explored as therapeutics for cancer treatments with a promising benefit.^[^ ^2^ ^]^ As a typical second messenger, Ca^2+^ can induce mitochondrial dysfunction and disrupt cell homeostasis by reducing mitochondrial membrane potentials, mitochondrial morphological changes, and mitochondrial respiratory disorders after its excess accumulation to result in apoptosis of cancer cells.^[^ ^3^ ^]^ Cyclic guanosine monophosphate‐adenosine monophosphate (cGAMP) is an essential second messenger in immune signaling as it can be used an agonist for stimulator of interferon genes (STING) pathway that is able to trigger antitumor T‐cell immunity.^[^ ^4^ ^]^ Thus, these second messengers provide potential options for cancer therapy via eliminating malignant cells by various mechanisms. However, they are often difficult to cross cellular plasma membrane and show poor accumulations in malignant cells and tumor sites, and thereby the therapeutic outcomes are limited.^[^ ^5^ ^]^ From another perspective, the overenrichment of these second messengers in healthy tissues without precise regulation will also cause some adverse effects.^[^ ^6^ ^]^ As a result, alternative strategies should be explored to further improve the therapeutic benefits and reduce adverse effects of second messengers.

Radiodynamic therapy (RDT) is a common clinical treatment option that can ablate tumors via generating reactive oxygen species (ROS) with X‐ray irradiation.^[^ ^7^ ^]^ Compared to chemotherapy, RDT shows the virtues of more effectiveness, especially for local lesions and lower systemic side effects.^[^ ^8^ ^]^ More importantly, RDT is able to overcome the tissue penetration obstacle of phototherapy, and thus permits treatments of deep‐seated tumors.^[^ ^9^ ^]^ Recently, RDT is widely used as an adjuvant to improve the efficacies of other therapies for synergistic actions.^[^ ^10^ ^]^ In addition to tumor ablation, RDT induces immunogenic cell death (ICD) to release different types of signal molecules for modulating the tumor immunosuppressive microenvironment and also achieve abscopal effect.^[^ ^11^ ^]^ For example, a nanoscale metal‐organic layer‐based nanoradiosensitizer was reported to mediate RDT for ICD induction and deliver cGAMP for STING activation, thus eliciting systemic immune response for tumor therapy.^[^ ^12^ ^]^

The purposeful delivery of therapeutics into local lesions is essential to improve the therapeutic benefits and reduce systematic side effects.^[^ ^13^ ^]^ Although tumor‐targeting nanocarriers can achieve this purpose by enhancing their enrichment in tumor cells, their uncontrolled release of drugs in normal tissues still should be concerned.^[^ ^14^ ^]^ Alternatively, stimulus‐responsive drug delivery nanoplatforms can ensure the on‐demand cargo releases in a more specific and selective manner for precision cancer therapy.^[^ ^15^ ^]^ Various stimuli including endogenous pH, ROS, and enzyme and exogenous light, X‐ray and ultrasound have enabled the generations of plentiful and versatile responsive nanosystems for the precision medicine.^[^ ^16^ ^]^ Particularly, X‐ray as an exogenous stimulus can overcome the poor specificity of endogenous ones and the tissue penetration concerns of light, which represents a promising tool to precisely control therapeutic delivery.^[^ ^17^ ^]^ Designs of X‐ray‐responsive nanosystems will contribute to improving therapeutic outcomes of second messengers by utilizing RDT as adjuvant and relieving the side effects by the controlled release way, which however has not been explored.

**Figure 1:**
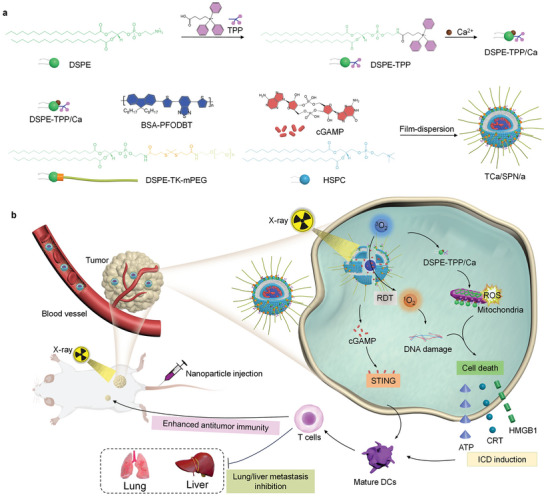
The X‐ray responsive second messenger release strategy by TCa/SPN/a for RDT‐combined tumor therapy. a) The schematic of fabrication processes of TCa/SPN/a. b) The schematic of X‐ray responsive second messenger release and RDT‐combined tumor therapy using TCa/SPN/a.

This study reports an X‐ray responsive second messenger release strategy by designing Ca^2+^‐ and cGAMP‐contained semiconducting polymer nanomessenger (TCa/SPN/a) for RDT‐combined tumor therapy. 1,2‐Distearoyl‐sn‐glycero‐3‐phosphorylethanolamine (DSPE)‐triphenylphosphonium (TPP) with Ca^2+^ chelation (DSPE‐TPP/Ca) as the mitochondria‐targeting Ca compound, a STING agonist (cGAMP), a semiconducting polymer and singlet oxygen (^1^O_2_)‐responsive 1,2‐distearoyl‐snglycero‐3‐phosphoethanolamine‐thioketal‐(polyethylene glycol) (DSPE‐TK‐PEG) are used to construct this nanomessenger (TCa/SPN/a) via the liposome formation courses (**Figure** **1**a). The semiconducting polymer served as both drug carrier and radiodynamic sensitizer, which was reported to have some unique merits, such as good biocompatibility, excellent optical properties and high stability.^[^ ^18^ ^]^ Under irradiation of X‐ray, semiconducting polymer acted as radiodynamic sensitizer to produce ROS, which would achieve on‐demand drug delivery via a responsive manner, thereby contributing to the enhanced therapeutic outcomes and reduced side effects. TCa/SPN/a displayed an effective enrichment in tumors, which enabled RDT to ablate tumors under X‐ray irradiation and induce tumor cell ICD. In addition, the generated ^1^O_2_ during RDT effect could destroy ^1^O_2_‐responsive DSPE‐TK‐PEG to allow activatable delivery of DSPE‐TPP/Ca and cGAMP. DSPE‐TPP/Ca targeted to mitochondria to deliver Ca^2+^ for ROS generation and mitochondria damage via calcium overload. As a STING agonist, cGAMP further boosted immunological effect following ICD induction (Figure 1b). After TCa/SPN/a injection and X‐ray irradiation, the growths of bilateral 4T1 tumors and tumor metastasis in mouse models were greatly inhibited, showing a satisfying therapeutic benefit.

## 2 Results and Discussion

### 2.1 Design and Characterization of TCa/SPN/a

To achieve mitochondria‐targeting effect of Ca^2+^ compound, mitochondria targeting ligand (TPP) was conjugated to DSPE to synthesize DSPE‐TPP. ^1^H NMR data could verify the successful synthesis (Figure S1, Supporting Information). The synthesized DSPE‐TPP was incubated in aqueous solution of CaCl_2_ to obtain DSPE‐TPP/Ca. Ca^2+^ showed a high binding effect with the phospholipid moiety within DSPE to allow Ca^2+^ ion chelation.^[^ ^19^ ^]^ DSPE‐TPP/Ca exhibited a peak at 191 nm, which was overlapped with Ca^2+^, verifying that Ca^2+^ was successfully loaded (Figure S2, Supporting Information). TCa/SPN/a were prepared through a liposome formation course of film‐dispersion and hydration using liposome excipient (HSPC), DSPE‐TK‐PEG, BSA‐PFODBT, cGAMP and DSPE‐TPP/Ca as the main components. Such a TCa/SPN/a showed a liposome structure containing water‐soluble BSA‐PFODBT and cGAMP in the inside core. Although hydrophobic PFODBT could be directly used to form nanoparticles, the formed structures were poorly stable and the loading efficacy was limited. Therefore, the water‐soluble BSA‐stabilized PFODBT (BSA‐PFODBT) was used for synthesis of TCa/SPN/a, which could improve the nanoparticle stability and loading efficacy. Other control samples including SPN, SPN/a, TCa/SPN were also prepared according to the similar methods.

**Figure 2:**
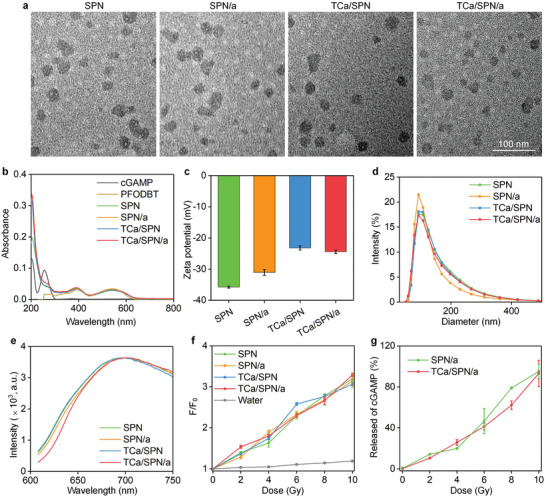
Preparation and characterization of SPN, SPN/a, TCa/SPN, and TCa/SPN/a. a) Appearance morphology images of SPN, SPN/a, TCa/SPN and TCa/SPN/a. b) Absorbance property of cGAMP, PFODBT, SPN, SPN/a, TCa/SPN and TCa/SPN/a. c) Zeta potentials of SPN, SPN/a, TCa/SPN and TCa/SPN/a (*n* = 3). d) Profiles of size distribution of SPN, SPN/a, TCa/SPN and TCa/SPN/a. e) Fluorescence spectra of SPN, SPN/a, TCa/SPN and TCa/SPN/a. f) Fluorescence intensity changes of SOSG probe (*F*/*F*_0_) of aqueous solutions containing SPN, SPN/a, TCa/SPN and TCa/SPN/a at different radiation doses (*n* = 3). g) The release efficiency of cGAMP for SPN/a and TCa/SPN/a under X‐ray irradiation of different doses (*n* = 3). Data are presented with mean ± SD.

SPN, SPN/a, TCa/SPN and TCa/SPN/a were uniform in size and regularly spherical in the TEM images, which verified the successful preparation of these four nanoparticles (**Figure** **2**a). The core–shell structure was not observed for these nanoparticles because of their small sizes and completely organic components.^[^ ^20^ ^]^ UV–vis results showed that SPN/a and TCa/SPN/a were loaded with cGAMP as the characteristic absorption peak of free cGAMP at 256 nm could be detected (Figure 2b). Meanwhile, SPN, SPN/a, TCa/SPN and TCa/SPN/a also had peaks at 390 and 540 nm, which were characteristic absorption peaks of PFODBT. The zeta potential of SPN and SPN/a was ‐35.8 ± 0.3 mV and ‐31.1 ± 0.9 mV, while TCa/SPN and TCa/SPN/a similarly exhibited a higher zeta potential, ‐23.2 ± 0.7 mV for TCa/SPN and ‐24.4 ± 0.5 mV for TCa/SPN/a (Figure 2c). The diameter of SPN, SPN/a, TCa/SPN and TCa/SPN/a was measured to be 90.6 ± 1.9, 87.1 ± 3.0, 88.3 ± 2.9, and 87.3 ± 3.34 nm, respectively (Figure 2d). The result indicated that these nanoparticles had similar diameters due to the same formation courses and the loadings of Ca^2+^ and cGAMP did not greatly affect the diameters. Meanwhile, the stability of these nanoparticles was illustrated as the particle diameters of these nanoparticles did not change conspicuously for 15 d (Figure S3, Supporting Information). Fluorescence spectral peaks of SPN, SPN/a, TCa/SPN and TCa/SPN/a were consistent (Figure 2e). The emission peaks could be manifestly observed at 700 nm, indicating that the fluorescence characteristics of PFODBT was not affected after formation of nanoparticles. SPN, SPN/a, TCa/SPN and TCa/SPN/a displayed an excellent capacity of ^1^O_2_ generation and the ^1^O_2_ production effect was increased with the irradiation dose (Figure 2f), which was verified using a ^1^O_2_ fluorescence probe (SOSG) as the indicator. Under the maximum X‐ray irradiation dose (10 Gy), fluorescence intensities of SOSG in SPN, SPN/a, TCa/SPN and TCa/SPN/a solutions were enhanced by at least 2.1 times. The significant increase of fluorescence signal of SOSG illustrated the rapid generation of ^1^O_2_. However, the SOSG fluorescence intensity change was unconspicuous for blank sample without nanoparticles.

The loading content and encapsulation efficiency of cGAMP in TCa/SPN/a was measured to be 2.0% and 94.1%, respectively. The ROS‐responsive release of cGAMP from SPN/a and TCa/SPN/a after X‐ray irradiation was studied. Under various irradiation doses, the release efficiency of cGAMP for TCa/SPN/a was 0.2%, 10.5%, 25.8%, 41.2%, 62.2%, and 93.1% (Figure 2g). The release efficiency of cGAMP from SPN/a was similar to that of TCa/SPN/a, which was 0.5%, 14.2%, 19.9%, 46.6%, 78.9% and 95.1%, respectively under X‐ray irradiation for 0, 2, 4, 6, 8, and 10 Gy, respectively. These results validated that TCa/SPN/a and SPN/a were activated by X‐ray for controlled release of cGAMP, which was due to the presence of cleavable amphiphilic polymers in their shells destroyed by the generated ^1^O_2_. Furthermore, the red blood cell hemolysis rates of all these nanoparticles were very low (Figure S4, Supporting Information), which verified their good blood compatibility for systemic administration.

### 2.2 In Vitro Therapeutic Efficacy and DNA Damage Evaluation

**Figure 3:**
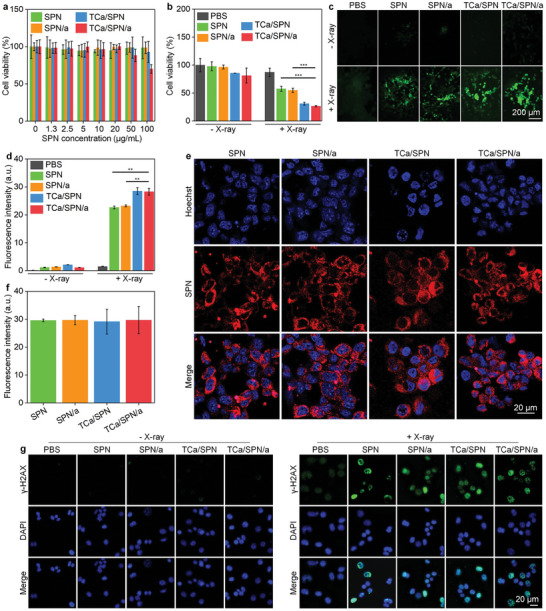
In vitro therapeutic efficacy and DNA damage studies. a) Cell viability analysis of SPN‐, SPN/a‐, TCa/SPN‐ and TCa/SPN/a‐treated 4T1 cells (*n* = 5). b) Cell viability analysis of 4T1 cancer cells after SPN, SPN/a, TCa/SPN and TCa/SPN/a incubations with or without X‐ray irradiation (*n* = 5). c) Fluorescence images of 4T1 cells after ROS generation. d) Quantitative analysis of the fluorescence intensity of the generated ROS (*n* = 5). e) The uptake efficacy of 4T1 cells in SPN, SPN/a, TCa/SPN and TCa/SPN/a groups. f) Quantitative analysis of the fluorescence signals of 4T1 cell images with nanoparticle uptake (*n* = 5). g) DNA damage analysis for 4T1 cells in SPN, SPN/a, TCa/SPN and TCa/SPN/a groups with or without X‐ray irradiation. Data are presented with mean ± SD, (**) *p* < 0.01, (***) *p* < 0.001, unpaired two‐tailed Student's t tests.

The biocompatibility of SPN, SPN/a, TCa/SPN and TCa/SPN/a was detected by utilizing 4T1 cancer cells, bone marrow stem cells (BMSCs), and NIH3T3 fibroblast cells. CCK‐8 analysis results revealed that all these nanoparticles did not cause significant cytotoxicity to 4T1 cancer cells and the normal cells (BMSCs and NIH3T3) after 24 h of incubation (**Figures** **3**a and S5, Supporting Information). Subsequently, 4T1 cells were irradiated with X‐ray and viability of 4T1 cells was obviously reduced, but the viability of treated cells without X‐ray irradiation did not change (Figure 3b). In the treatment groups, the cell viability of 4T1 cells in TCa/SPN + X‐ray (31.0 ± 2.5%) and TCa/SPN/a + X‐ray (26.6 ± 1.1%) groups was similar, which was much lower than that of SPN + X‐ray (57.6 ± 4.6%) and SPN/a + X‐ray (54.9 ± 3.9%) groups. This verified that the Ca^2+^ loading could significantly enhance the cancer cell killing effect for TCa/SPN and TCa/SPN/a. Next, a ROS fluorescence probe was utilized to detect the ROS levels after X‐ray irradiation of the treated 4T1 cells (Figure 3c). ROS signals were found in SPN + X‐ray, SPN/a + X‐ray, TCa/SPN + X‐ray, and TCa/SPN/a + X‐ray groups. The results of quantitative ROS analysis showed that the signal intensity in SPN + X‐ray, SPN/a + X‐ray, TCa/SPN + X‐ray, and TCa/SPN/a + X‐ray groups was significantly increased (Figure 3d). The ROS generation in TCa/SPN + X‐ray and TCa/SPN/a + X‐ray groups via RDT effect and calcium overloading was higher relative to that in SPN + X‐ray and SPN/a + X‐ray groups by only RDT. In TCa/SPN/a‐treated cells, the ROS signals and intensities were found to elevate as the increase of X‐ray doses (Figure S6, Supporting Information), verifying that the intracellular ROS generation was depended on the doses of X‐ray irradiation.

Fluorescence images of SPN‐, SPN/a‐, TCa/SPN‐ and TCa/SPN/a‐incubated cells were captured to study phagocytosis effects. The red fluorescence signal of PFODBT (nanoparticles) was similarly detected inside 4T1 cells (Figure 3e). The quantitative results showed that the uptake effect of SPN, SPN/a, TCa/SPN and TCa/SPN/a was not significantly different, which may be due to the fact that they had a similar nanoparticle surface (Figure 3f). Subsequently, flow cytometry analysis results showed that the cells of SPN, SPN/a, TCa/SPN and TCa/SPN/a groups had much stronger fluorescence intensity compared with PBS group, further confirming that these nanoparticles were effectively swallowed by cancer cells (Figure S7, Supporting Information). The levels of DNA damage with X‐ray irradiation were verified by detecting the phosphorylated form of histone H2AX. The fluorescence signals of γ‐H2AX in SPN + X‐ray, SPN/a + X‐ray, TCa/SPN + X‐ray and TCa/SPN/a + X‐ray groups were much stronger than that in PBS + X‐ray group and other groups without X‐ray irradiation (Figure 3g and Figure S8, Supporting Information). These results suggested that ROS produced by these nanoparticles with X‐ray radiation could induce a large number of DNA double‐strand breaks. In addition, cGAMP functioning as a STING agonist did not have obvious influences on cell viability and ROS generation for in vitro cancer cells.

### 2.3 ICD Effect and Mitochondrial Damage Analysis In Vitro

**Figure 4:**
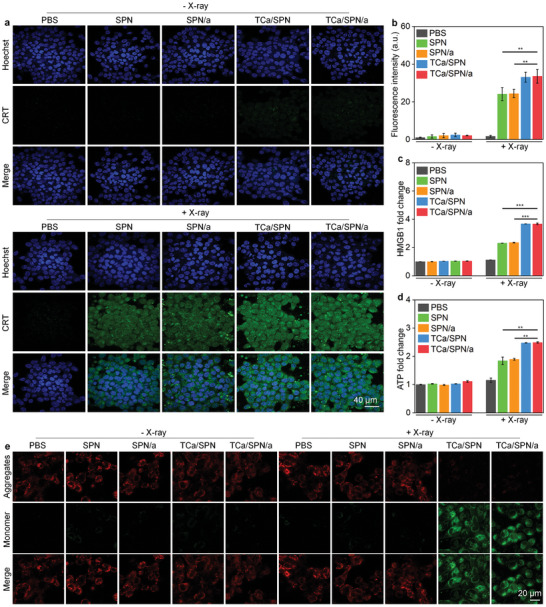
ICD effect and mitochondrial damage analysis in vitro. a) The detection of CRT for treated 4T1 cells via immunofluorescence staining analysis. b) Quantitative analysis of CRT staining images (*n* = 3). c) HMGB1 level analysis for treated 4T1 cells (*n* = 3). d) Extracellular ATP level analysis for the treated 4T1 cells (*n* = 3). e) CLSM images of JC‐1 aggregates (red) and JC‐1 monomer (green) in 4T1 cells after SPN, SPN/a, TCa/SPN and TCa/SPN/a incubation with or without X‐ray irradiation. Data are presented with mean ± SD, (**) *p* < 0.01, (***) *p* < 0.001, unpaired two‐tailed Student's t tests.

Both RDT effect and Ca^2+^‐based ROS generation could induce ICD of tumor cells, which was characterized by high expression of CRT accompanied by HMGB1 release from nucleus and excess ATP secretion.^[^ ^21^ ^]^ As shown in **Figure** **4**a, SPN, SPN/a, TCa/SPN and TCa/SPN/a treatment after X‐ray irradiation could promote the high expression of CRT in cells. TCa/SPN + X‐ray and TCa/SPN/a + X‐ray groups were observed to have stronger fluorescence signals of CRT compared to SPN + X‐ray and SPN/a + X‐ray groups. CRT signal in TCa/SPN + X‐ray and TCa/SPN/a + X‐ray groups was about 1.4‐fold stronger than that of SPN + X‐ray and SPN/a + X‐ray groups and 19.0‐fold stronger relative to that in PBS + X‐ray group (Figure 4b). In addition, TCa/SPN + X‐ray and TCa/SPN/a + X‐ray groups exhibited an enhanced releasing level of HMGB1 from the nucleus compared with SPN + X‐ray and SPN/a + X‐ray groups (Figure 4c). Compared with PBS + X‐ray group, the HMGB1 secretion level in SPN +X‐ray and SPN/a +X‐ray groups was increased by 2.1‐fold, while which in TCa/SPN + X‐ray and TCa/SPN/a + X‐ray groups were increased by 3.3‐fold (Figure 4c). Higher ATP releasing levels for cells in TCa/SPN + X‐ray and TCa/SPN/a + X‐ray groups were also observed, which were about 2.1‐fold higher than that in PBS + X group (Figure 4d). The ATP levels in SPN + X‐ray and SPN/a + X‐ray groups were only increased by approximately 1.6‐fold. These results suggested that TCa/SPN/a + X‐ray could significantly promote the ICD of tumor cells.

JC‐1 probe was applied to verify membrane potential changes and mitochondrial damages. When the released DSPE‐TPP/Ca targeted to mitochondria, calcium overloading caused ROS generation in mitochondria to achieve membrane potential changes and mitochondrial damages. Red fluorescent signal represented aggregate forms of normal mitochondrial membrane, while the green fluorescent signal indicated monomer forms of the abnormal mitochondrial membrane. The green fluorescence signals were observed for cells in TCa/SPN + X‐ray and TCa/SPN/a + X‐ray groups, but red signals could be found in all other groups (Figure 4e). Green fluorescence signal of TCa/SPN + X‐ray and TCa/SPN/a + X‐ray groups was at least 16.3‐fold stronger than that in all other groups (Figure S9, Supporting Information). All findings verified mitochondria damages by TCa/SPN and TCa/SPN/a after X‐ray irradiation.

### 2.4 Evaluation of Tumor Accumulation and ICD Effect

**Figure 5:**
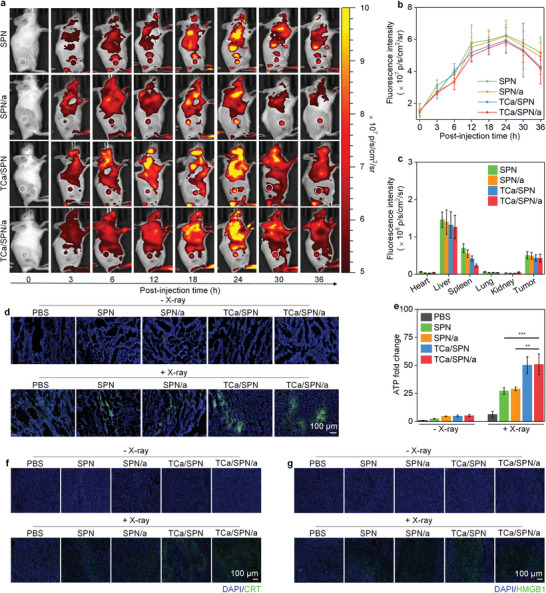
Evaluation of tumor accumulation and ICD effect in vivo. a) The accumulation analysis of SPN, SPN/a, TCa/SPN and TCa/SPN/a in tumors sites via IVIS spectrum imaging system (the tumor areas were represented by white dotted circles). b) Fluorescence intensity analysis of the tumor sites at certain post‐injection time point (*n* = 3). c) Fluorescence intensity of tumor tissues and organs (*n* = 3). d) CLSM images of the ROS generation in tumor site after treatments (*n* = 3). e) Determination of ATP release levels in tumors (*n* = 3). f) Immunofluorescence images of the tumors for evaluating the expression of CRT after treatments. g) Immunofluorescence images of the tumors for evaluating the expression of HMGB1 after treatments. Data are presented with mean ± SD, (**) *p* < 0.01, (***) *p* < 0.001, unpaired two‐tailed Student's t tests.

Determining the maximum accumulation time of nanoparticles at tumor sites and distribution of major organs were of great significance for the follow‐up treatments. SPN, SPN/a, TCa/SPN and TCa/SPN/a were injected to tumor mouse models via tail vein and fluorescence signals of tumor sites were detected at different time points. The fluorescence signals at the tumor site of all groups of mice gradually increased with the extension of time, which reached the maximum signal values at 24 h (**Figure** **5**a). The maximal signal values at 24 h were approximately 3.7‐fold higher than that at 0 h (Figure 5b), confirming the accumulation effect of nanoparticles in tumor tissues. After 36 h of post‐injection, the mice were dissected to obtain major organs for fluorescence imaging to observe biodistribution. Obvious fluorescence signals were observed in tumors, spleen, and liver for injected mice (Figure S10, Supporting Information). Quantitative results of the major organs showed that the liver accumulation was higher compared to that of spleen and tumors (Figure 5c). Thus, SPN, SPN/a, TCa/SPN and TCa/SPN/a not only showed a prominent enrichment effect at tumor area, but also accumulated into liver and spleen, which was due to the existence of reticuloendothelial system in liver and spleen, such as high‐density blood vessels and phagocytes.^[^ ^22^ ^]^

The ROS generation and ICD effect of tumors were confirmed to ensure their therapeutic actions. By using a ROS fluorescence probe, the manifest green fluorescence signal of ROS could be observed in SPN + X‐ray, SPN/a + X‐ray, TCa/SPN + X‐ray and TCa/SPN/a + X‐ray groups, but no obvious fluorescence signal was observed in these groups without X‐ray irradiation (Figure 5d). The analysis of fluorescence intensity showed that the ROS levels of TCa/SPN + X‐ray and TCa/SPN/a + X‐ray groups were about 1.2‐fold higher than that of SPN + X‐ray and SPN/a + X‐ray groups and the fluorescence intensity was at least 6.5‐fold higher compared to that of PBS + X‐ray group (Figure S11, Supporting Information). Furthermore, ATP, CRT, and HMGB1 were evaluated successively to verify the ICD effect at the tumor level after treatments. ATP secreting levels for SPN + X‐ray, SPN/a + X‐ray, TCa/SPN + X‐ray, and TCa/SPN/a + X‐ray groups were obviously increased compared to control group (Figure 5e). ATP release of SPN +X‐ray and SPN/a +X‐ray groups was increased by about 27.2‐fold, which in TCa/SPN + X‐ray and TCa/SPN/a + X‐ray groups was approximately 50.3‐fold higher than that of PBS group. Immunofluorescence staining of tumors indicated that the expression of CRT was upregulated in SPN + X‐ray, SPN/a + X‐ray, TCa/SPN + X‐ray, and TCa/SPN/a + X‐ray groups (Figure 5f and Figure S12, Supporting Information). There were obvious fluorescence signals of HMGB1 in SPN + X‐ray, SPN/a + X‐ray, TCa/SPN + X‐ray and TCa/SPN/a + X‐ray groups (Figure 5g). The levels of HMGB1 expression in SPN + X‐ray, SPN/a + X‐ray, TCa/SPN + X‐ray and TCa/SPN/a + X‐ray groups were 43.2 ± 3.0, 48.0 ± 6.1, 66.1 ± 5.3 and 67.0 ± 7.0‐fold higher than that in PBS group, respectively (Figure S13, Supporting Information). All the ATP, CRT and HMGB1 data indicated that TCa/SPN/a with X‐ray irradiation could induced a more pronounced ICD effect of tumors.

### 2.5 Antitumor and Anti‐Metastasis Analysis

**Figure 6:**
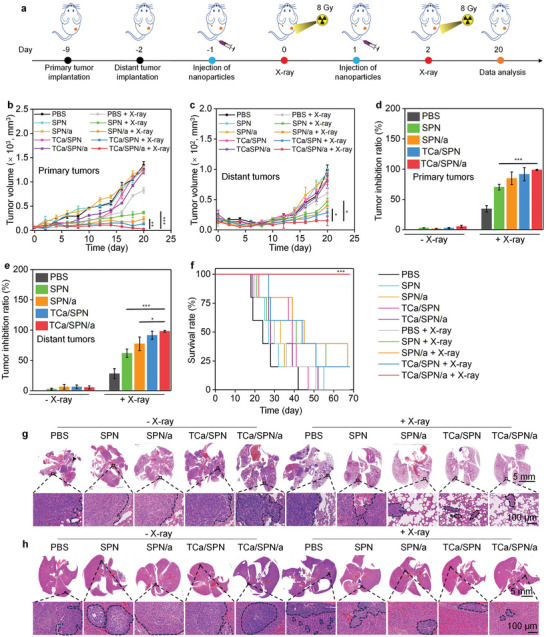
Antitumor and anti‐metastasis analysis. a) Strategies of bilateral tumor model construction, nanoparticle injection, X‐ray irradiation of primary tumors, and data analysis. b) The volume curves of primary tumors in SPN‐, SPN/a‐, TCa/SPN‐ and TCa/SPN/a‐treated mice with or without X‐ray irradiation (*n* = 6). c) The volume curves of distant tumors (*n* = 6). d) Analysis of tumor inhibition rates for primary tumors (*n* = 6). e) Analysis of tumor inhibition rates for distant tumors (*n* = 6). f) The survival curves of treated mice (*n* = 5). g) H&E staining images of isolated lungs in each group. h) H&E staining images of isolated livers in different treatment groups. Data are presented with mean ± SD, (*) *p* < 0.05, (**) *p* < 0.01, (***) *p* < 0.001, unpaired two‐tailed Student's t tests.

Tumor mouse models were divided into ten groups to evaluate the antitumor and anti‐metastasis efficacy of nanomessengers with X‐ray irradiation, including PBS, SPN, SPN/a, TCa/SPN, TCa/SPN/a, PBS + X‐ray, SPN + X‐ray, SPN/a + X‐ray, TCa/SPN + X‐ray and TCa/SPN/a + X‐ray groups. The whole course of treatment was carried out in sequence according to the original plan (**Figure** **6**a). The antitumor effects were evaluated by observing tumor growths and survival rate of mice. In SPN, SPN/a, TCa/SPN, TCa/SPN/a treated groups without X‐ray irradiation, the tumor volumes showed a steady upward trend that was similar to PBS and PBS + X‐ray groups, while the rates of tumor volume increase slowed significantly for SPN + X‐ray, SPN/a + X‐ray, TCa/SPN + X‐ray and TCa/SPN/a + X‐ray groups (Figure 6b,c). On day 20, the tumor growths of primary tumors for PBS, SPN, SPN/a, TCa/SPN and TCa/SPN/a groups were roughly the same, which were increased by 22.0 ± 1.2‐, 20.5 ± 0.6‐, 18.3 ± 0.9‐, 20.5 ± 0.2‐ and 18.9 ± 0.8‐fold, respectively, indicating that the antitumor properties of nanomessengers without X‐ray irradiation were weak. In SPN + X‐ray, SPN/a + X‐ray, TCa/SPN + X‐ray and TCa/SPN/a + X‐ray groups, the tumor volumes of primary tumors decreased significantly. Tumor volume in SPN + X‐ray and SPN/a + X‐ray groups was increased by 5.3 ± 0.4‐ and 3.5 ± 0.7‐fold, respectively, however which in TCa/SPN + X‐ray and TCa/SPN/a + X‐ray groups was only increased by 2.0 ± 0.3‐ and 0.5 ± 0.1‐fold. Similar results were found for distant tumors and the tumor growth inhibition in TCa/SPN/a + X‐ray group was the highest. The body weights of treated mice had a slight increase during the monitoring period (Figure S14, Supporting Information). The tumor inhibition rate for primary tumors of SPN + X‐ray, SPN/a + X‐ray, TCa/SPN + X‐ray and TCa/SPN/a + X‐ray groups was 70.3 ± 4.6%, 84.9 ± 2.3%, 91.6 ± 1.7% and 98.8 ± 0.9%, respectively (Figure 6d). The tendency of tumor suppression rates for distant tumors was similar to those of primary tumors and the highest tumor suppression rate was found in TCa/SPN/a + X‐ray group (Figure 6e). Synergistic effect (Q value) was calculated according to a previous work,^[^ ^23^ ^]^ which was 1.37 and 1.53 for primary and distant tumors in TCa/SPN/a + X‐ray group, respectively, indicating the synergism action. The mouse survival rate still remained 100% in TCa/SPN/a + X‐ray group for 68 days, while the survival rate of all other groups was no more than 40% (Figure 6f). Hematoxylin and eosin (H&E) staining was applied for histologic analysis to further verify the antitumor effect. The staining images revealed the several necrotic areas of primary and distant tumors in SPN + X‐ray, SPN/a + X‐ray, TCa/SPN + X‐ray and TCa/SPN/a + X‐ray groups (Figure S15, Supporting Information). The necrotic areas of tumors in TCa/SPN/a + X‐ray group were the largest, further confirming the best antitumor effect. Additionally, these treatments were found to show negligible damage to normal organs of mice (Figure S16, Supporting Information). The blood routine and biochemical analysis showed the normal index parameters for blood samples of mice in all treatment groups (Figures S17 and S18, Supporting Information).

Encouraged by the prominent therapeutic effect, the anti‐metastasis efficacy of TCa/SPN/a with X‐ray irradiation was evaluated. After a 30 d treatment course, the lung and liver tissues were used for H&E staining to analyze lung and liver metastases. For the high metastasis of normal organs, large lung metastases were observed in PBS, SPN, SPN/a, TCa/SPN, TCa/SPN/a and PBS + X‐ray groups, while the areas of lung metastasis nodules in SPN + X‐ray, SPN/a + X‐ray, TCa/SPN + X‐ray and TCa/SPN/a + X‐ray groups were reduced to some extent (Figure 6g). TCa/SPN/a + X‐ray group had the smallest metastatic nodule area in lung that was at least 2.1‐fold lower than that in SPN + X‐ray, SPN/a + X‐ray and TCa/SPN + X‐ray groups (Figure S19, Supporting Information). Similarly, liver metastatic nodules were the most evident in PBS, SPN, SPN/a, TCa/SPN, TCa/SPN/a and PBS + X‐ray groups, but a few metastatic nodules were observed in liver of SPN + X‐ray, SPN/a + X‐ray and TCa/SPN + X‐ray groups (Figure 6h). The average liver metastatic area in TCa/SPN/a + X‐ray group was much lower than that in SPN + X‐ray, SPN/a + X‐ray and TCa/SPN + X‐ray groups (Figure S20, Supporting Information). This verified the excellent anti‐metastasis efficacy of TCa/SPN/a with X‐ray irradiation.

### 2.6 Evaluation of Immunotherapy Effect

To verify the roles of ICD and the released cGAMP in immunotherapy course, the maturation of dendritic cells (DCs) was analyzed. The matured DC levels in SPN + X‐ray, SPN/a + X‐ray, TCa/SPN + X‐ray and TCa/SPN/a + X‐ray groups were increased compared to those in PBS group (Figure S21, Supporting Information). The matured DC level in TCa/SPN/a + X‐ray group was 74.9 ± 4.1%, which was 1.8‐, 1.7‐ and 1.3‐fold higher as compared with SPN + X‐ray, SPN/a + X‐ray and TCa/SPN + X‐ray groups, respectively. This confirmed the promoted maturation of DCs for immunotherapy.

**Figure 7:**
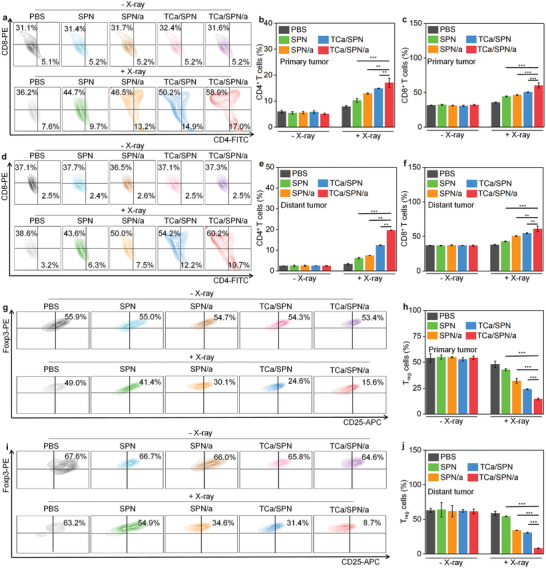
Evaluation of immunotherapy effect. a) The ratios of CD4^+^ and CD8^+^ T cells in CD3^+^ cells in primary tumor sites via flow cytometry analysis. The proportion analysis of b) CD4^+^ T cells and c) CD8^+^ T cells (*n* = 5). d) The ratios of CD4^+^ and CD8^+^ T cells in CD3^+^ cells in distant tumor sites via flow cytometry analysis. The proportion analysis of e) CD4^+^ T cells and f) CD8^+^ T cells (*n* = 5). g) The levels of T_reg_ cells in primary tumor sites via flow cytometry analysis. h) The proportion analysis of T_reg_ cells in primary tumors (*n* = 5). i) The levels of T_reg_ cells in distant tumor sites via flow cytometry analysis. j) The proportion analysis of T_reg_ cells in distant tumors (*n* = 5). Data are presented with mean ± SD, (**) *p* < 0.01, (***) *p* < 0.001, unpaired two‐tailed Student's t tests.

Then primary and distant tumors of treated mice were extracted for analysis of CD4^+^ and CD8^+^ T cell levels. For primary tumors, SPN + X‐ray, SPN/a + X‐ray, TCa/SPN + X‐ray and TCa/SPN/a + X‐ray significantly increased the proportions of CD4^+^ and CD8^+^ T cells (**Figure** **7**a). In SPN + X‐ray, SPN/a + X‐ray, TCa/SPN + X‐ray and TCa/SPN/a + X‐ray groups, the proportions of CD4^+^ T cells increased successively, which reached 10.3 ± 0.7%, 13.0 ± 0.3%, 14.9 ± 0.2% and 17.1 ± 1.8%, respectively (Figure 7b). For CD8^+^ T cells, the proportion of TCa/SPN/a + X‐ray group was 60.3 ± 3.6%, which was higher than that of SPN + X‐ray (44.5 ± 0.8%), SPN/a + X‐ray (46.5 ± 0.6%) and TCa/SPN + X‐ray (50.5 ± 0.5%) groups (Figure 7c). Similarly, there was a significant increase in the proportions of CD4^+^ and CD8^+^ T cells for distant tumors in the treated groups (Figure 7d). The level of CD4^+^ T cells in distant tumors of TCa/SPN/a + X‐ray group was the highest (19.6 ± 0.1%), but which in other groups was only 2.4 ± 0.1% to 12.4 ± 0.2% (Figure 7e). The proportion of CD8^+^ T cells in distant tumors of TCa/SPN/a + X‐ray group was also the highest (61.1 ± 4.0%), which was around 1.9‐fold (37.1 ± 0.3%) higher as compared to PBS group (Figure 7f). Apparently, the immune effects of distant tumors were consistent with those of primary tumors. These results confirmed that TCa/SPN/a + X‐ray showed the highest efficacy in promoting immune T cell activation. This superior immune response for TCa/SPN/a + X‐ray group over other control groups may be due to dual roles of STING activation and ICD effect. Additionally, the proportions of immunosuppressive T_reg_ cells were assessed. The proportions of T_reg_ cells in primary tumors of SPN + X‐ray (42.8 ± 1.0%), SPN/a + X‐ray (32.0 ± 2.3%), TCa/SPN + X‐ray (24.0 ± 0.5%) and TCa/SPN/a + X‐ray (14.5 ± 0.9%) groups were significantly reduced compared to that in PBS group (Figure 7g,h). Similarly, the proportions of T_reg_ cells in distant tumors of SPN + X‐ray, SPN/a + X‐ray, TCa/SPN + X‐ray and TCa/SPN/a + X‐ray groups were also decreased (Figure 7i). The T_reg_ cell population of TCa/SPN/a + X‐ray group was only 8.2 ± 0.5%, lower than that of SPN + X‐ray (54.2 ± 0.7%), SPN/a + X‐ray (34.1 ± 0.3%) and TCa/SPN + X‐ray (30.7 ± 0.9%) groups (Figure 7j). All above results suggested that TCa/SPN/a + X‐ray treatment obviously decreased the levels of T_reg_ cells to promote immunotherapy.

The combination of RDT and immunotherapy has been reported in previous studies,^[^ ^7^, ^24^ ^]^ while the designed TCa/SPN/a was different from these previous nanomedicine platforms. Two second messengers, Ca^2+^ and cGAMP were integrated into TCa/SPN/a and their releases could be activated by X‐ray irradiation, which improved the therapeutic efficacy via synergistic effect. The loaded Ca^2+^ and cGAMP could mediate ROS generation, enhanced ICD effect, and immune response activation for promoting RDT and immunotherapy.

## 3 Conclusion

This study has presented a Ca^2+^ and cGAMP‐contained semiconducting polymer nanomessenger (TCa/SPN/a) for effective and precise cancer therapy. The final TCa/SPN/a exhibited similar particle morphology, colloidal stability, optical properties, biocompatibility and tumor accumulating efficacy as their control counterparts. Two key therapeutic elements (Ca^2+^ and cGAMP) could be released by TCa/SPN/a in a X‐ray‐activatable manner, which was achieved via destroying nanoparticle structures due to the generation of ^1^O_2_ by the RDT effect of semiconducting polymer (PFODBT). In addition, TCa/SPN/a plus X‐ray irradiation could ablate tumors and induce ICD effect for improving the tumor immunogenicity that boosted the immunotherapy efficacy. Ca^2+^ and cGAMP also exerted therapeutic functions after on‐demand releases via causing mitochondria damage and activating STING pathway, respectively. Such a novel regimen was found to obviously inhibit the growths of bilateral tumors and also abolish tumor metastasis in metastatic 4T1 tumor mouse models. This nanomessenger should be the first second messenger‐contained nanomedicine system with X‐ray‐activatable cargo release and therapeutic actions for tumor precision medicine. Further integrations of tumor‐targeting ligands will allow enrichment of more nanomessengers into tumor sites, providing alternative options for cancer therapy with amplified benefits and reduced adverse effects.

## 4 Experimental Section

### Synthesis of DSPE‐TPP and DSPE‐TPP/Ca

EDC (43.13 mg), NHS (26.07 mg), and TPP (32.19 mg) were dissolved in 3 mL chloroform for stirring at 40 °C for 2 h. Next, DSPE (11.22 mg) dissolved in 1 mL chloroform was mixed with the TPP for stirring at 40 °C for 3 d. The synthesized DSPE‐TPP was purified via dialysis and freeze‐dried for ^1^H NMR analysis. The DSPE‐TPP (10.5 mg) dissolved in THF was mixed with aqueous solution of CaCl_2_ (1 mg) for stirring at room temperature for 2 h. Subsequently, the samples were dialyzed using a 500 Da dialysis bag to remove free Ca^2+^. After purification, DSPE‐TPP/Ca was freeze‐dried to obtain powder.

### Synthesis of BSA‐PFODBT

PFODBT (0.25 mg) dissolved in THF was mixed with 10 mL BSA solution (water/THF = 9:1 of volume, 0.5 mg BSA) and the mixture was sonicated for 30 min. Next, THF was removed to obtain BSA‐PFODBT.

### Synthesis of TCa/SPN/a

DSPE‐TPP/Ca (10 mg), DSPE‐TK‐mPEG (10 mg), and HSPC (10 mg) were dissolved in 1 mL chloroform, respectively and mixed at room temperature under sonication. The chloroform was evaporated at 40 °C for 1 h to form a lipid film. BSA‐PFODBT solution and 0.2 mL cGAMP aqueous solution (1 mg mL^−1^) were mixed with the lipid film for stirring at 60 °C. Next, the solution was cooled to room temperature and then sonicated for 40 min at a power of 120 W. The lipid solution was first extruded through a 400 nm pore polycarbonate membrane and then extruded through a 200 nm pore polycarbonate membrane. The solution was then purified and concentrated via ultrafiltration to obtain TCa/SPN/a. SPN was synthesized via similar steps using HSPC, DSPE‐mPEG and BSA‐PFODBT. SPN/a was synthesized using HSPC, DSPE‐mPEG, BSA‐PFODBT and cGAMP. TCa/SPN was synthesized using HSPC, DSPE‐mPEG, DSPE‐TPP/Ca, and BSA‐PFODBT.

### Measurement of ^1^O_2_ Generation

The ^1^O_2_ indicator (SOSG) was used to detect the ^1^O_2_ generation for SPN, SPN/a, TCa/SPN and TCa/SPN/a solutions (20 µg mL^−1^). The solutions were treated by 0, 2, 4, 6, 8, and 10 Gy of X‐ray with 100 kV energy voltage. After X‐ray irradiation, the solutions were measured using fluorescence spectrophotometer to detect the fluorescence intensity at 525 nm. *F*/*F*_0_ was calculated to verify the ^1^O_2_ generation (*F*_0_ was the initial fluorescence intensity, and *F* were the fluorescence intensities after treatments).

### The cGAMP Release Efficacy Analysis

TCa/SPN/a and SPN/a solutions (50 µg mL^−1^) were irradiated by X‐ray (100 kV energy voltage). After X‐ray irradiation, the solutions were filtrated through 70 µm filter screen and the filtrate was collected for detection of cGAMP using HPLC (the stetting liquidity parameters: 25% methanol + 75% ultrapure water).

### Hemolysis Assay

Fresh blood was taken from the orbital veins of BALB/c mice and the upper serum was removed to gained red cells. After rinsing with 1 × PBS at least 5 times to remove the plasma completely, red blood cell diluent was obtained by adding 10 mL 1 × PBS. Next, the concentrations of SPN, SPN/a, TCa/SPN or TCa/SPN/a were adjusted to 3.1, 6.3, 12.5, 25, 50 and 100 µg mL^−1^. Red blood cells were treated by various solutions for 2 h for calculating the hemolysis rate.

### Establishment of Tumor Mouse Models

All procedures were approved by the Institutional Ethics Committee for Animal Experimentation of Donghua University. To establish tumor mouse models, 4T1 cell suspension was implanted into right flank of BALB/c female mice (2 × 10^6^ cells for each mouse) as the primary tumors. After 7 d, left flank of mice was also implanted in same number of cells to establish the distant tumors. In this period of time, the growths of the tumors were observed to estimate tumor volumes. When volumes reached around 100 mm^3^, the relevant animal experiments were conducted.

### Analysis of Tumor Accumulation

BALB/c mice with 4T1 tumors were injected with SPN, SPN/a, TCa/SPN and TCa/SPN/a (300 µg mL^−1^, 200 µL per each one) via tail vein. At the definite time point (0, 3, 6, 12, 18, 24, 30, and 36 h), the fluorescence images of the tumor sites were captured. After determining the time points of maximum accumulation, the mice were euthanized to collect major organs, and IVIS spectrum imaging system was used to obtain the fluorescence images. Living Image 4.3 Software was used for the fluorescence intensity analysis.

### Intratumoral ^1^O_2_ Generation Analysis

The tumor mouse models were administrated with PBS, SPN, SPN/a, TCa/SPN or TCa/SPN/a (300 µg mL^−1^, 200 µL per each one) via tail vein. After 24 h, H_2_DCFH‐DA working solution (10 µm) was injected in situ into the primary tumors, and the tumor sites were irradiated by 8 Gy of X‐ray. After treatments, the tumors were extracted for DAPI staining and fluorescence signals were observed using CLSM.

### Analysis of Intratumoral ICD Biomarkers

The tumor mouse models were administrated with PBS, SPN, SPN/a, TCa/SPN or TCa/SPN/a (300 µg mL^−1^, 200 µL per each one) via tail vein and then the tumor sites were irradiated by 8 Gy of X‐ray. The tumor tissues of treated mice were extracted and homogenized to prepare suspension. The contents of ATP in suspension were determined using ATP detection kit. The extracted tumor tissues were used for CRT and HMGB1 level analysis via immunofluorescence staining.

### Blood Biochemical and Routine Analysis

After 1 d of each treatment, the blood samples were collected from the mice. The upper serum of blood was prepared for blood biochemical analysis and the lower red blood cells were used for blood routine analysis.

### In Vivo Tumor Inhibition Effect Evaluation

The mice for in vivo antitumor experiments included ten groups: PBS, SPN, SPN/a, TCa/SPN, TCa/SPN/a, PBS + X‐ray, SPN + X‐ray, SPN/a + X‐ray, TCa/SPN + X‐ray, TCa/SPN/a + X‐ray (300 µg mL^−1^, 200 µL per each mouse, 8 Gy of X‐ray irradiation). Nanoparticle injection and X‐ray irradiation were repeated twice at a one‐day interval, respectively. Tumor dimensions were recorded to calculate tumor volumes. Weight changes of mice were also recorded for 20 d. The tumors were collected for antitumor effect evaluation via section staining. The survival of treated mice was recorded for 68 d, and the Kaplan‐Meier survival curve of mice was described.

### Evaluation of Metastasis Inhibition

Mice were treated for 30 d and lung and liver were collected after treatments. The metastasis inhibition efficacies were evaluated via H&E staining of lung and liver tissues.

### Immune Response Analysis

4T1 tumor‐bearing mice were treated and tumor‐draining lymph nodes (TDLNs) were separated to homogenize in PBS solution. Single cell suspension was obtained after filtration by 70 µm strainers, followed by staining with BV786‐CD45, FITC‐CD11c, PE‐CD80 and APC‐CD86 at 4 °C for 40 min. Primary and distant tumors were isolated to prepare single cell suspension. Red blood cell lysis buffer was utilized to remove red blood cells and then single cell suspensions were added into lymphocyte separation solution to obtain lymphocytes. T cells staining regiments were BV421‐DAPI, BV786‐CD45, APC/Cyanine7‐CD3, FITC‐CD4 and PE‐CD8 antibodies. In addition, the staining scheme of T_reg_ cells was listed as follows: APC/Cyanine7‐CD3, FITC‐CD4, APC‐CD25, PE‐Foxp3 antibodies and Zombie Aqua dye. All the antibodies were diluted by approximate 100 times. BV421‐DAPI staining was performed to exclude dead cells. These samples were analyzed by flow cytometry.

### Statistical Analysis

The data involved in the experiment are expressed as mean ± standard deviation of the results with the sample size (n) for statistical analysis. The unpaired two‐tailed Student's t tests were adopted for data processing and analysis. The (*) *p* < 0.05, (**) *p* < 0.01 and (***) *p* < 0.001 indicated the statistical significance. All statistical analysis was performed using GraphPad Prism 8.0.

## Conflict of Interest

The authors declare no conflict of interest.

## Supporting information



Supporting Information

## Data Availability

The data that support the findings of this study are available from the corresponding author upon reasonable request.

## References

[1] a) Y. Oe , X. Wang , T. Patriarchi , A. Konno , K. Ozawa , K. Yahagi , H. Hirai , T. Tsuboi , T. Kitaguchi , L. Tian , Nat. Commun. 2020, 11, 471; 31980655 10.1038/s41467-020-14378-x PMC6981284

[2] a) P. Zheng , J. Ding , Asian J. Pharm. Sci. 2022, 17, 1; 35261641 10.1016/j.ajps.2021.10.004 PMC8888138

[3] a) L. Xu , G. Tong , Q. Song , C. Zhu , H. Zhang , J. Shi , Z. Zhang , ACS Nano 2018, 12, 6806; 29966081 10.1021/acsnano.8b02034

[4] a) D. Shae , K. W. Becker , P. Christov , D. S. Yun , A. K. Lytton‐Jean , S. Sevimli , M. Ascano , M. Kelley , D. B. Johnson , J. M. Balko , Nat. Nanotechnol. 2019, 14, 269; 30664751 10.1038/s41565-018-0342-5 PMC6402974

[5] a) S. T. Koshy , A. S. Cheung , L. Gu , A. R. Graveline , D. J. Mooney , Adv. Biosyst. 2017, 1, 1600013; 30258983 10.1002/adbi.201600013 PMC6152940

[6] a) Y. Xiao , Z. Li , A. Bianco , B. Ma , Adv. Funct. Mater. 2023, 33, 2209291;

[7] a) Z. Xu , T. Luo , J. Mao , C. McCleary , E. Yuan , W. Lin , Angew. Chem., Int. Ed. 2022, 61, e202208685; 10.1002/anie.202208685 PMC9647855 36149753

[8] a) Z. Yang , X. Ren , L. Li , J. Zhang , X. Yang , Y. Zhang , A. K. Whittaker , B. Yang , T. Wang , Q. Lin , Biomaterials 2025, 313, 122814; 39243672 10.1016/j.biomaterials.2024.122814

[9] a) J. Huang , L. Su , C. Xu , X. Ge , R. Zhang , J. Song , K. Pu , Nat. Mater. 2023, 22, 1421; 37667071 10.1038/s41563-023-01659-1

[10] a) J. Li , Z. You , S. Zhai , J. Zhao , K. Lu , ACS Appl. Mater. Interfaces 2023, 15, 21941; 37099714 10.1021/acsami.3c02361

[11] a) T. Liu , P. Pei , W. Shen , L. Hu , K. Yang , Small Methods 2023, 7, 2201401; 10.1002/smtd.202201401 36811166

[12] T. Luo , G. T. Nash , X. Jiang , X. Feng , J. Mao , J. Liu , A. Juloori , A. T. Pearson , W. Lin , Adv. Mater. 2022, 34, 2110588. 10.1002/adma.202110588 PMC9529854 35952624

[13] a) M. J. Mitchell , M. M. Billingsley , R. M. Haley , M. E. Wechsler , N. A. Peppas , R. Langer , Nat. Rev. Drug Discovery 2021, 20, 101; 33277608 10.1038/s41573-020-0090-8 PMC7717100

[14] a) Y. Zhang , Y. Wang , A. Zhu , N. Yu , J. Xia , J. Li , Angew. Chem., Int. Ed. 2024, 63, e202310252; 10.1002/anie.202310252 38010197

[15] a) Y. Zhang , J. Li , K. Pu , Biomaterials 2022, 291, 121906; 36395660 10.1016/j.biomaterials.2022.121906

[16] a) J. Wagner , D. e. Gößl , N. Ustyanovska , M. Xiong , D. Hauser , O. Zhuzhgova , S. Hocevar , B. l. Taskoparan , L. Poller , S. Datz , ACS Nano 2021, 15, 4450; 33648336 10.1021/acsnano.0c08384

[17] a) C. Yang , Y. Yang , Y. Li , Q. Ni , J. Li , J. Am. Chem. Soc. 2022, 145, 385; 36542856 10.1021/jacs.2c10177

[18] J. Li , J. Rao , K. Pu , Biomaterials 2018, 155, 217. 29190479 10.1016/j.biomaterials.2017.11.025 PMC5978728

[19] Z. Li , Y. Jiang , H. Zhao , L. Liu , Langmuir 2022, 38, 6612. 35578744 10.1021/acs.langmuir.2c00464

[20] X. Bai , Q. Chen , F. Li , Y. Teng , M. Tang , J. Huang , X. Xu , X.‐Q. Zhang , Nat. Commun. 2024, 15, 6844. 39122711 10.1038/s41467-024-51056-8 PMC11315999

[21] a) N. Guo , K. Ni , T. Luo , G. Lan , A. Arina , Z. Xu , J. Mao , R. R. Weichselbaum , M. Spiotto , W. Lin , ACS Nano 2021, 15, 17515; 34709030 10.1021/acsnano.1c04363

[22] J. Llop , T. Lammers , ACS Nano 2021, 15, 16974. 34748314 10.1021/acsnano.1c09139 PMC7612708

[23] H. Cai , R. Wang , X. Guo , M. Song , F. Yan , B. Ji , Y. Liu , Mol. Pharmaceutics 2021, 18, 2495. 10.1021/acs.molpharmaceut.0c01225 34078087

[24] K. Lu , C. He , N. Guo , C. Chan , K. Ni , G. Lan , H. Tang , C. Pelizzari , Y.‐X. Fu , M. T. Spiotto , Nat. Biomed. Eng. 2018, 2, 600. 31015630 10.1038/s41551-018-0203-4

