# Supplementary material for: Ca2+‐ and cGAMP‐Contained Semiconducting Polymer Nanomessengers for Radiodynamic‐Activated Calcium Overload and Immunotherapy
Source: Adv Sci (Weinh). 2024 Dec 16;12(6):2411739. doi: 10.1002/advs.202411739 (PMC11809400; doi:10.1002/advs.202411739)
Supplement: Supplementary file 1 — Supporting Information [file ADVS-12-2411739-s001.docx]

Supporting Information

Ca^2+^- and cGAMP-Contained Semiconducting Polymer Nanomessengers for Radiodynamic-Activated Calcium Overload and Immunotherapy

Danling Cheng, Libai Luo, Qin Zhang, Zheming Song, Yiduo Zhan, Wenzhi Tu*, Jingchao Li*, Qiming Ma*, Xianchang Zeng*

D. Cheng, Prof. X. Zeng

Institute of Immunology, Zhejiang University School of Medicine, Hangzhou, 310009, China

E-mail: xczeng@zju.edu.cn

Dr. L. Luo

Oncology Chemotherapy Department, Affiliated Hospital of Youjiang Medical University for Nationalities and Key Laboratory of Research on Clinical Molecular Diagnosis for High Incidence Diseases in Western Guangxi, Baise, China

Dr. Q. Zhang

Institute of Translational Medicine, Shanghai University, Shanghai 200444, China

Z. Song, Y. Zhan, Prof. J. Li

State Key Laboratory for Modification of Chemical Fibers and Polymer Materials, College of Biological Science and Medical Engineering, Donghua University, Shanghai 201620, China

E-mail: jcli@dhu.edu.cn

Dr. W. Tu

Department of Radiation Oncology, Shanghai General Hospital, Shanghai Jiao Tong University School of Medicine, Shanghai, 201620, China

E-mail: wenzhi.tu@shgh.cn

Prof. Q. Ma

Department of general surgery, The First Affiliated Hospital of Gannan Medical University, Ganzhou, 341000, China

E-mail: mqmgdwk1993@163.com

# 1. Supporting Figures


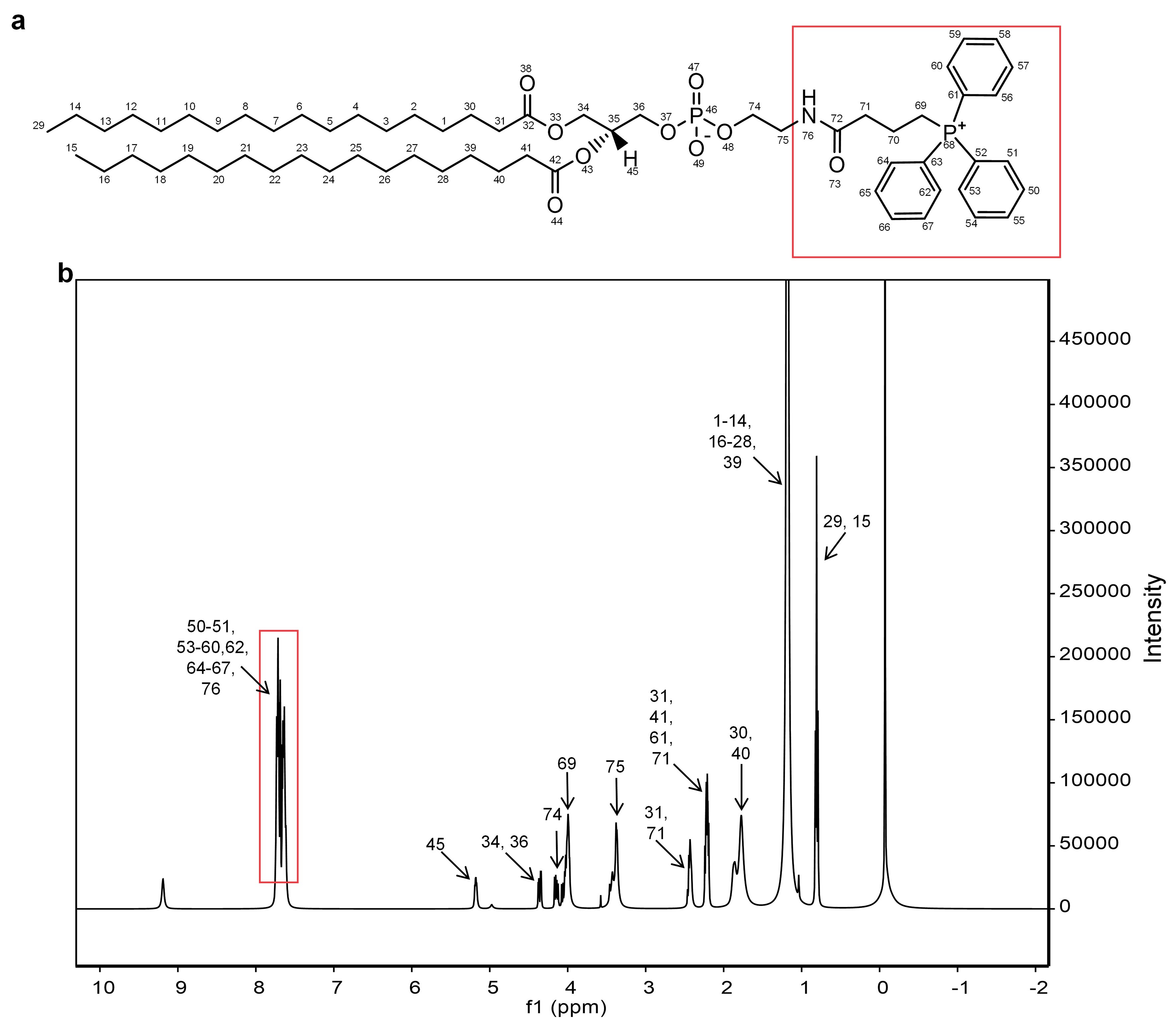


**Figure S1.** (a) Chemical structure of DSPE-TPP. (b) ^1^H NMR spectrum of DSPE-TPP in CDCl_3_.


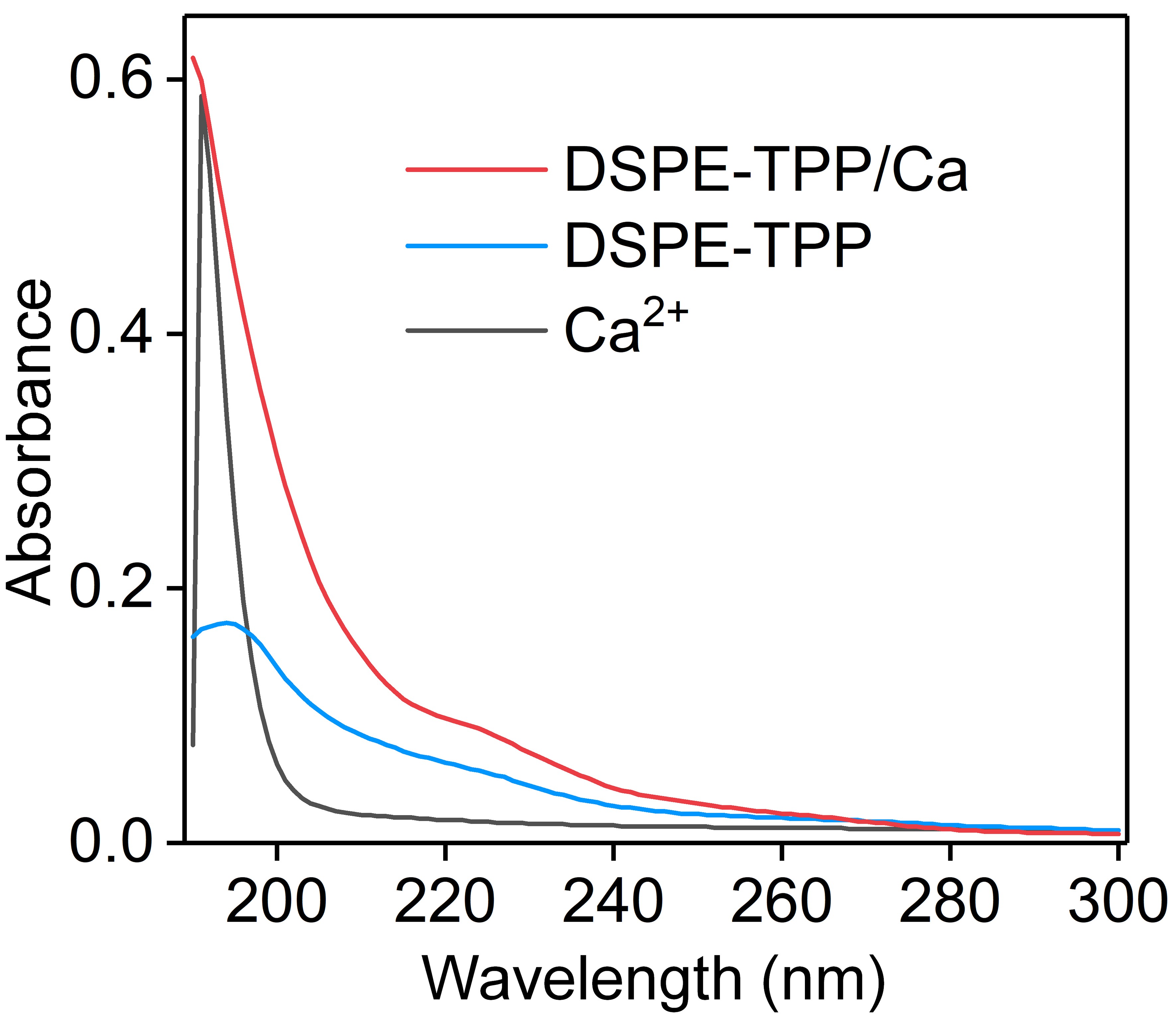


**Figure S2.** UV-vis absorption spectra of DSPE-TPP/Ca, DSPE-TPP and Ca^2+^ solutions.


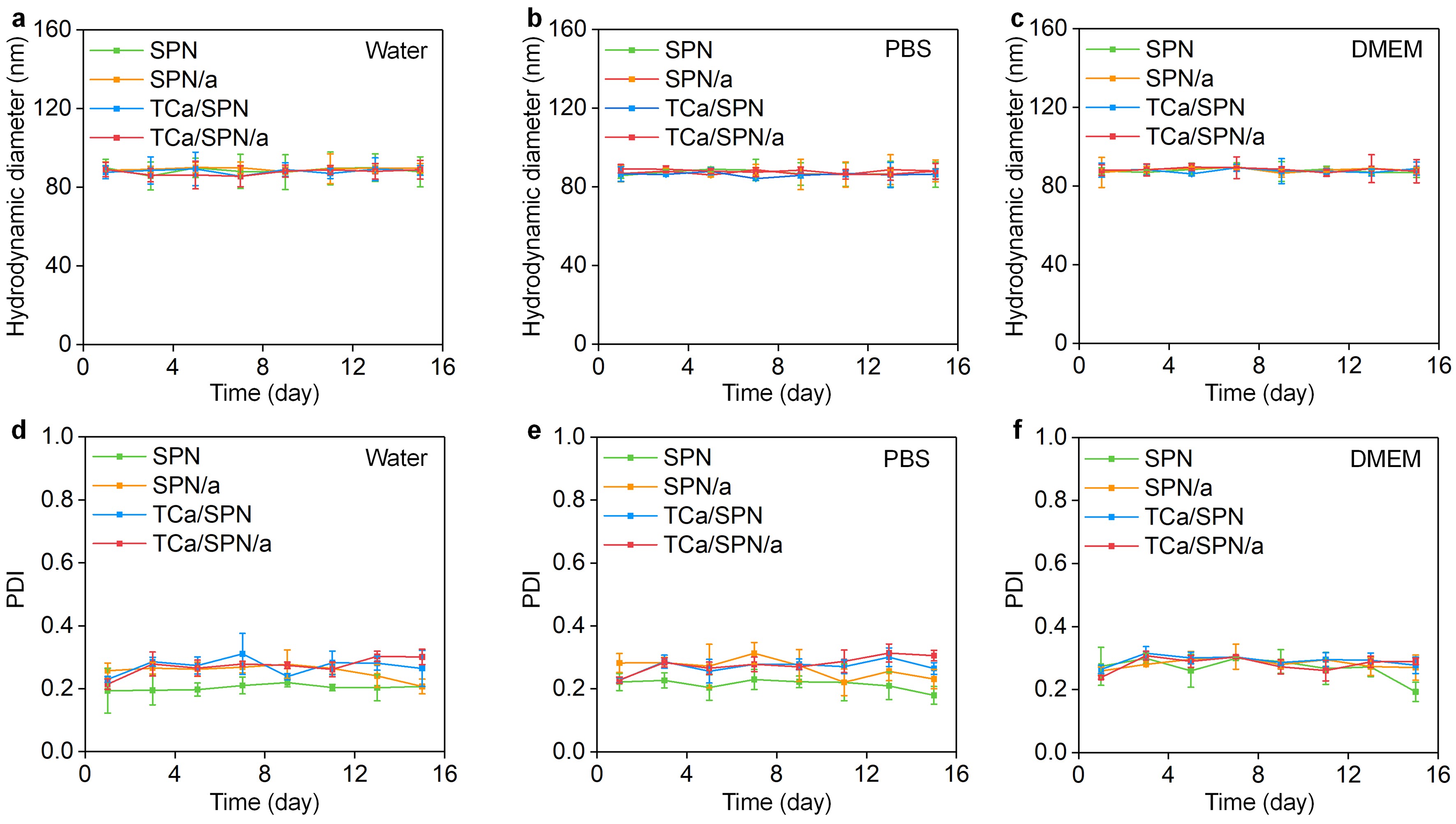


**Figure S3.** Particle size stability evaluation of SPN, SPN/a, TCa/SPN and TCa/SPN/a for 1, 3, 5, 7, 9, 11, 13 and 15 days in (a) water, (b) PBS and (c) DMEM medium (n = 5). The PDI values of SPN, SPN/a, TCa/SPN and TCa/SPN/a in (d) water, (e) PBS and (f) DMEM medium (n = 5). Data are presented with mean ± SD.


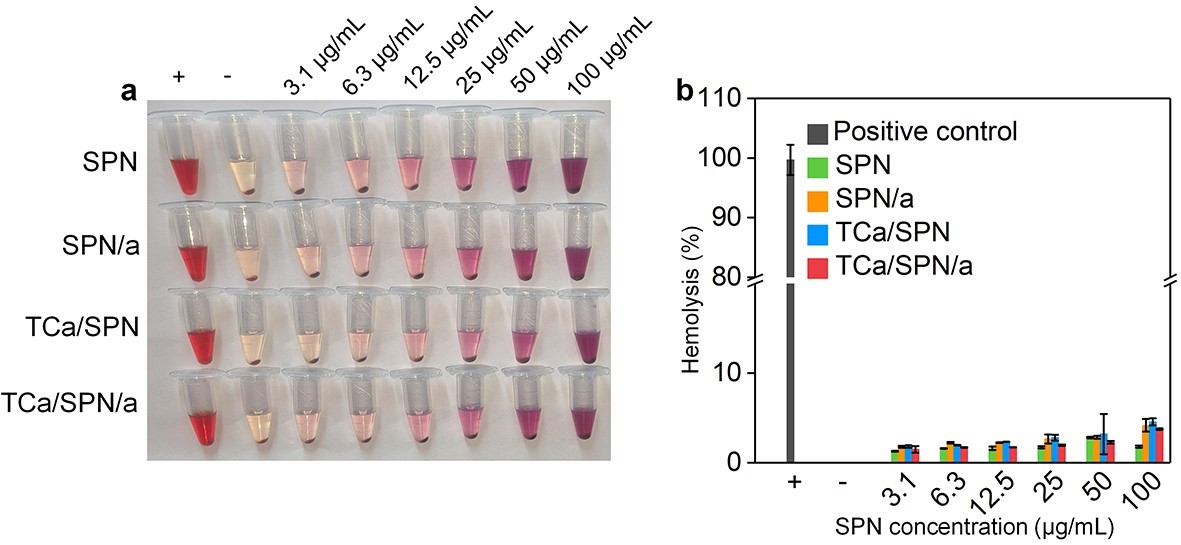


**Figure S4.** (a) The photos of red blood cells in positive control (water), negative control (PBS), SPN, SPN/a, TCa/SPN and TCa/SPN/a groups after centrifugation. (b) Hemolysis rates after treatment with positive control (water), negative control (PBS), SPN, SPN/a, TCa/SPN and TCa/SPN/a in various SPN concentrations (n = 3). Data are presented with mean ± SD.


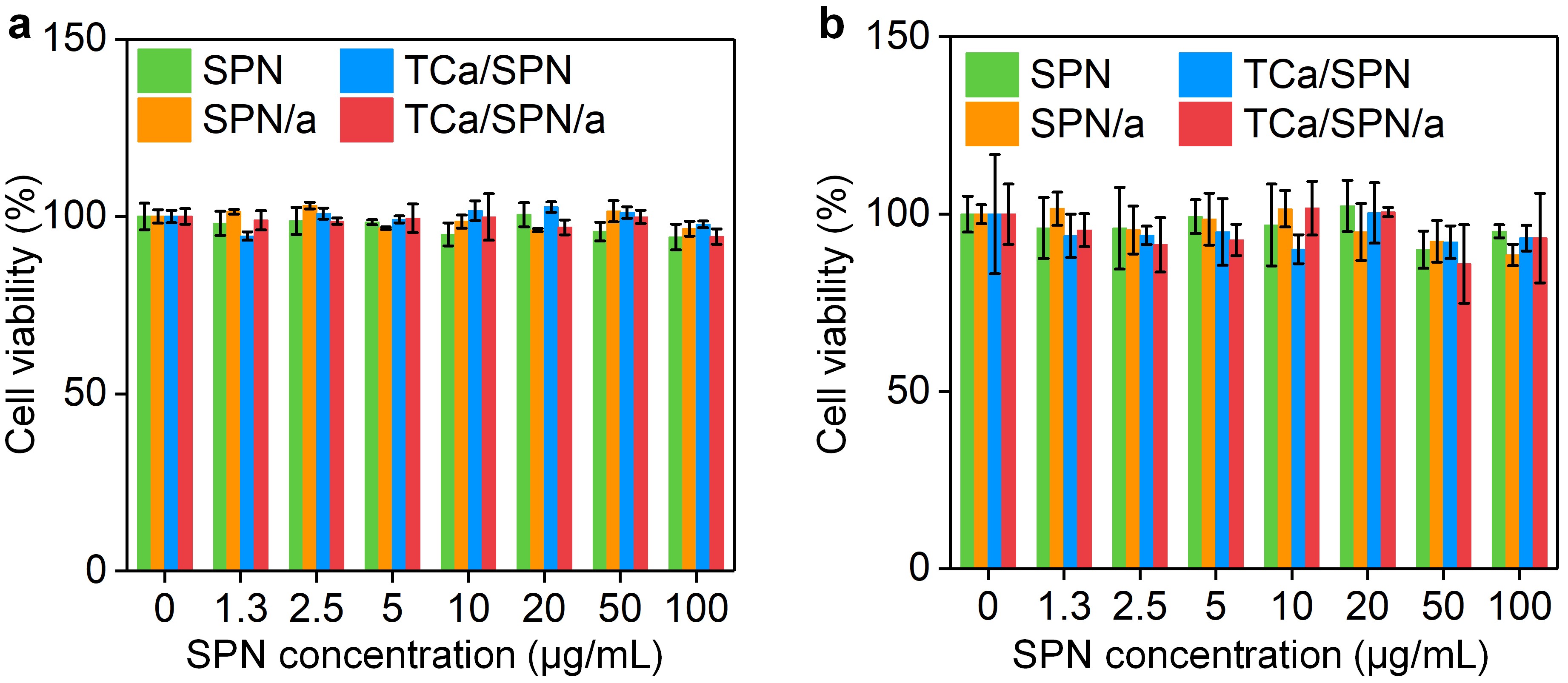


**Figure S5.** Viability analysis of (a) BMSCs and (b) NIH3T3 fibroblast cells after SPN, SPN/a, TCa/SPN and TCa/SPN/a treatments for 24 h (n = 5). Data are presented with mean ± SD.


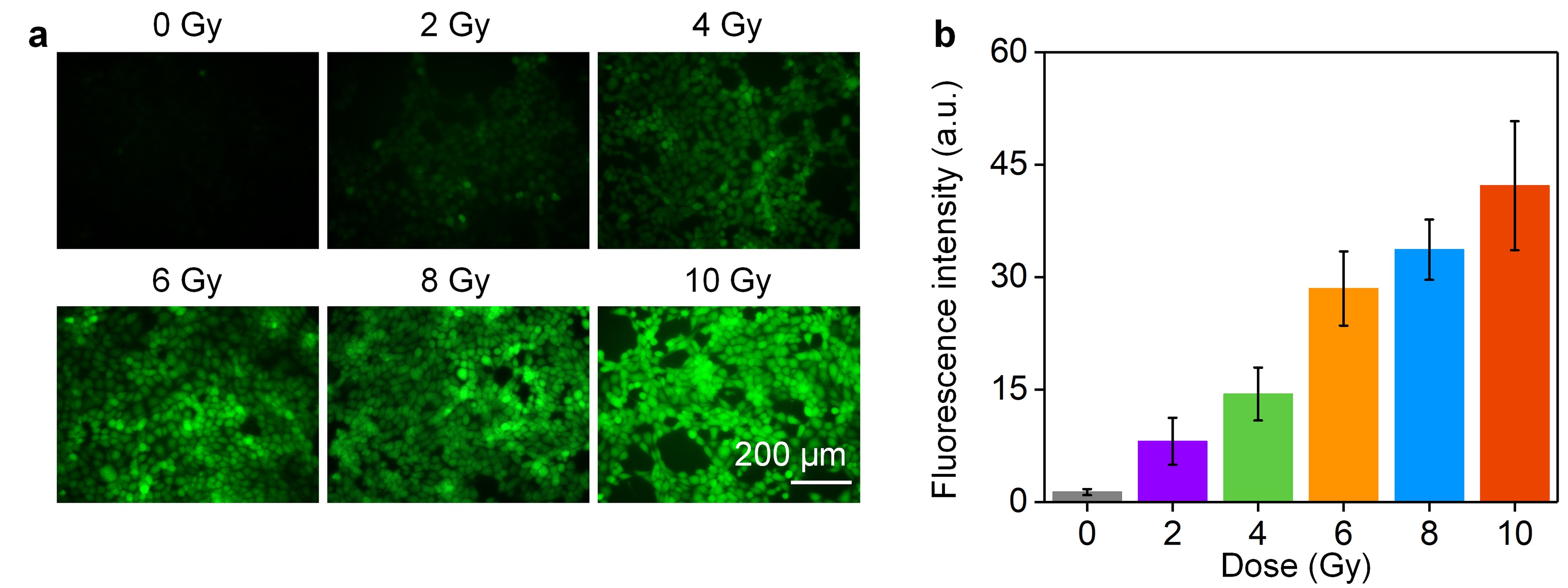


**Figure S6.** (a) ROS generation images for TCa/SPN/a-treated cells with X-ray irradiation at different doses. (b) The fluorescence intensity of ROS signals for TCa/SPN/a-treated cells with X-ray irradiation at different doses (n = 5). Data are presented with mean ± SD.


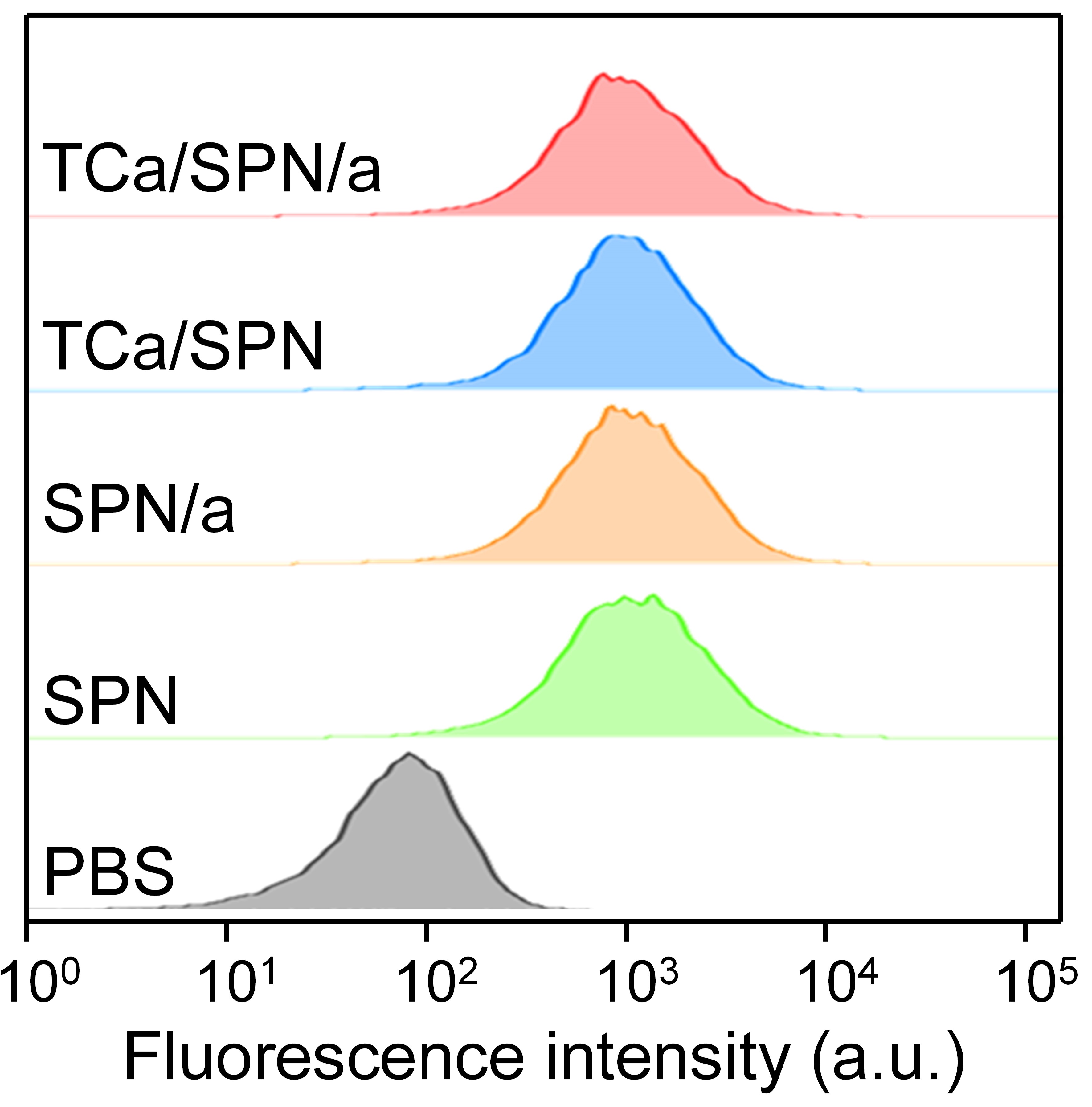


**Figure S7.** Fluorescence intensities of the treated 4T1 cells via FACS analysis.


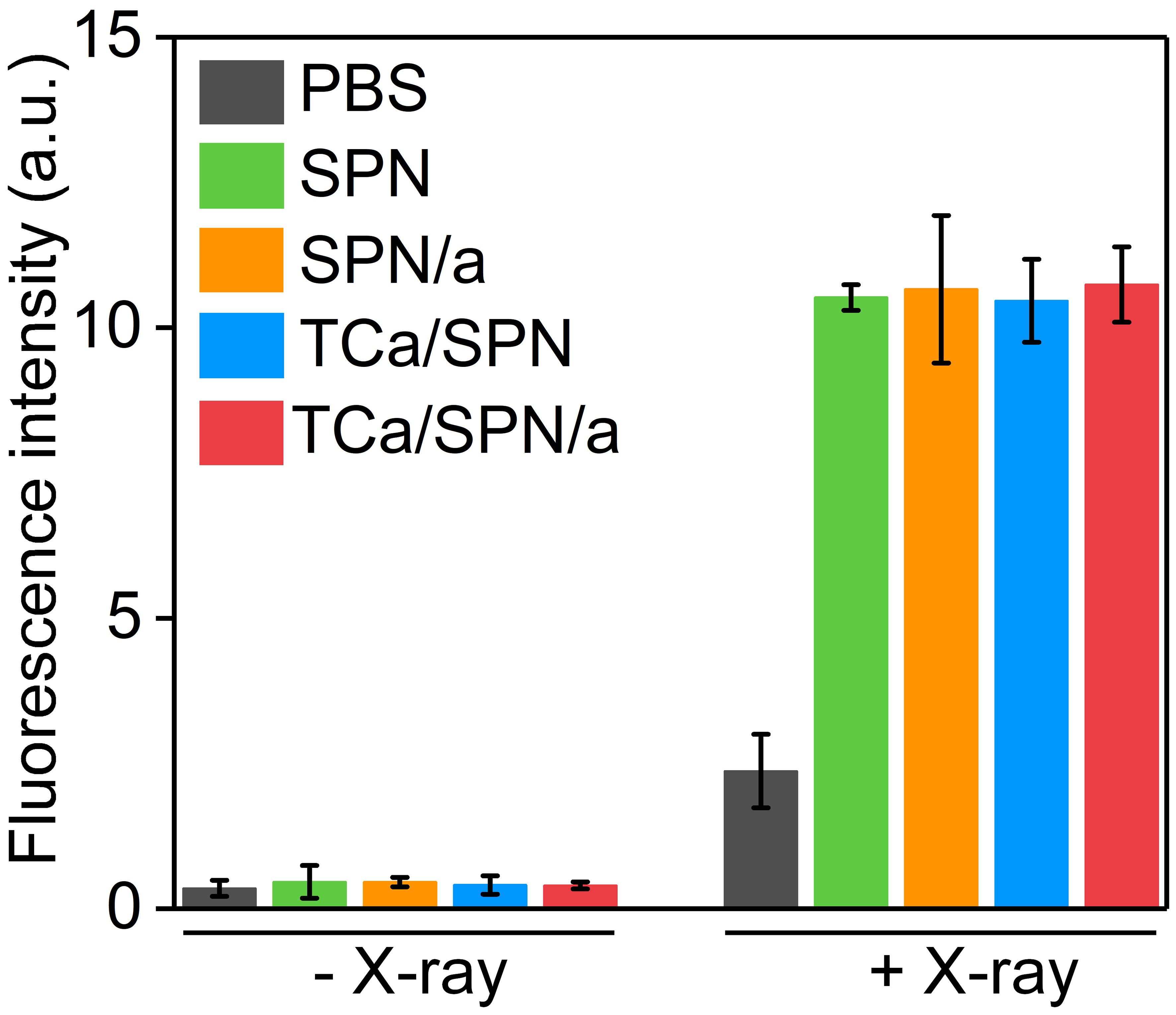


**Figure S8.** Quantitative analysis of fluorescence intensity of γ-H2AX content in treated cells (n = 5). Data are presented with mean ± SD.


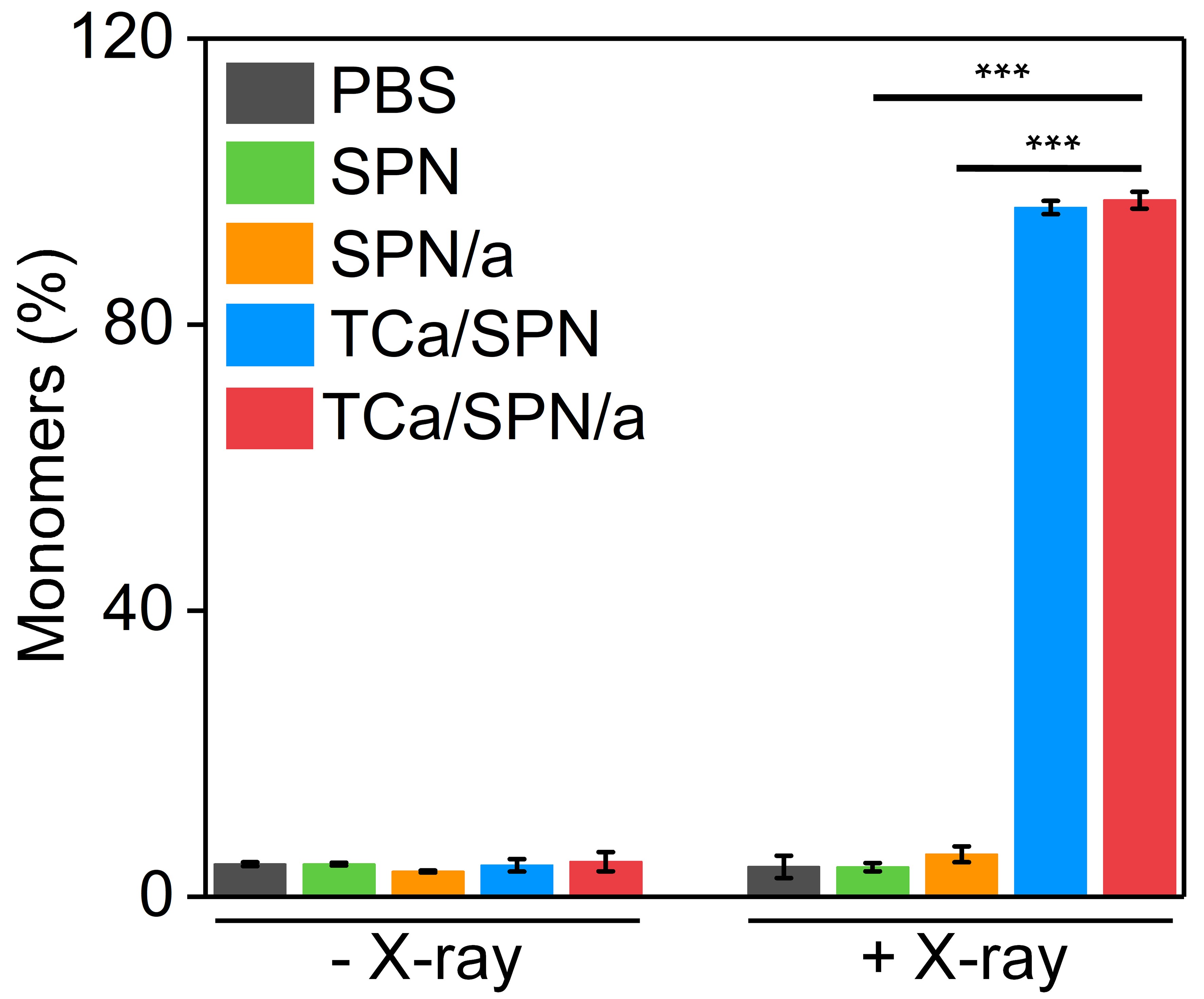


**Figure S9.** Analysis of the percentages of JC-1 monomer (green signal) of mitochondrial (n = 5). Data are presented with mean ± SD, (***) p < 0.001, unpaired two-tailed Student’s t tests.


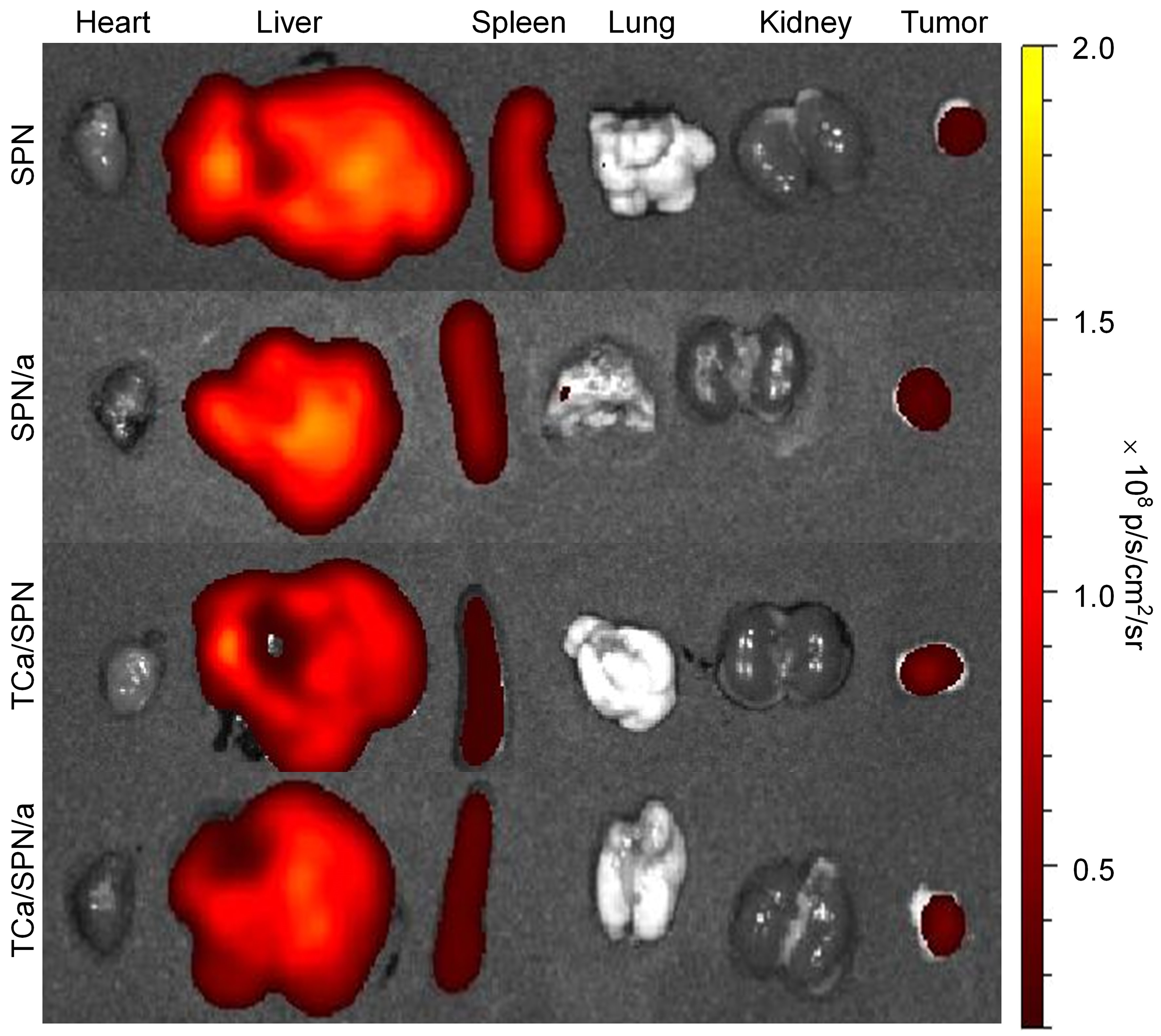


**Figure S10.** Fluorescence images of tumor tissues and major organs of mice at 36 h after intravenous injection of SPN, SPN/a, TCa/SPN and TCa/SPN/a.


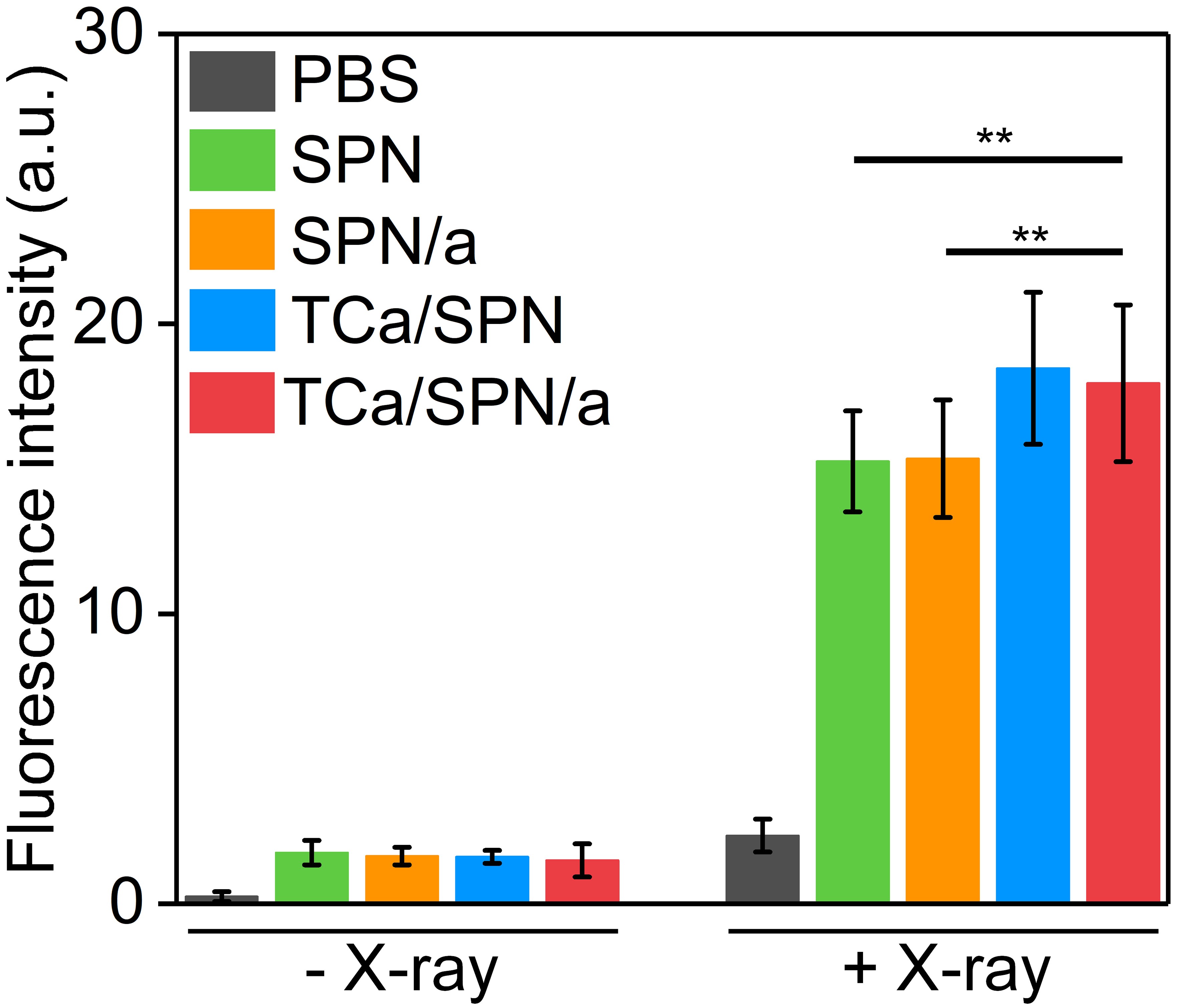


**Figure S11.** Quantitative analysis of fluorescence intensity of ROS in tumor regions (n = 5). Data are presented with mean ± SD, (**) p < 0.01, unpaired two-tailed Student’s t tests.


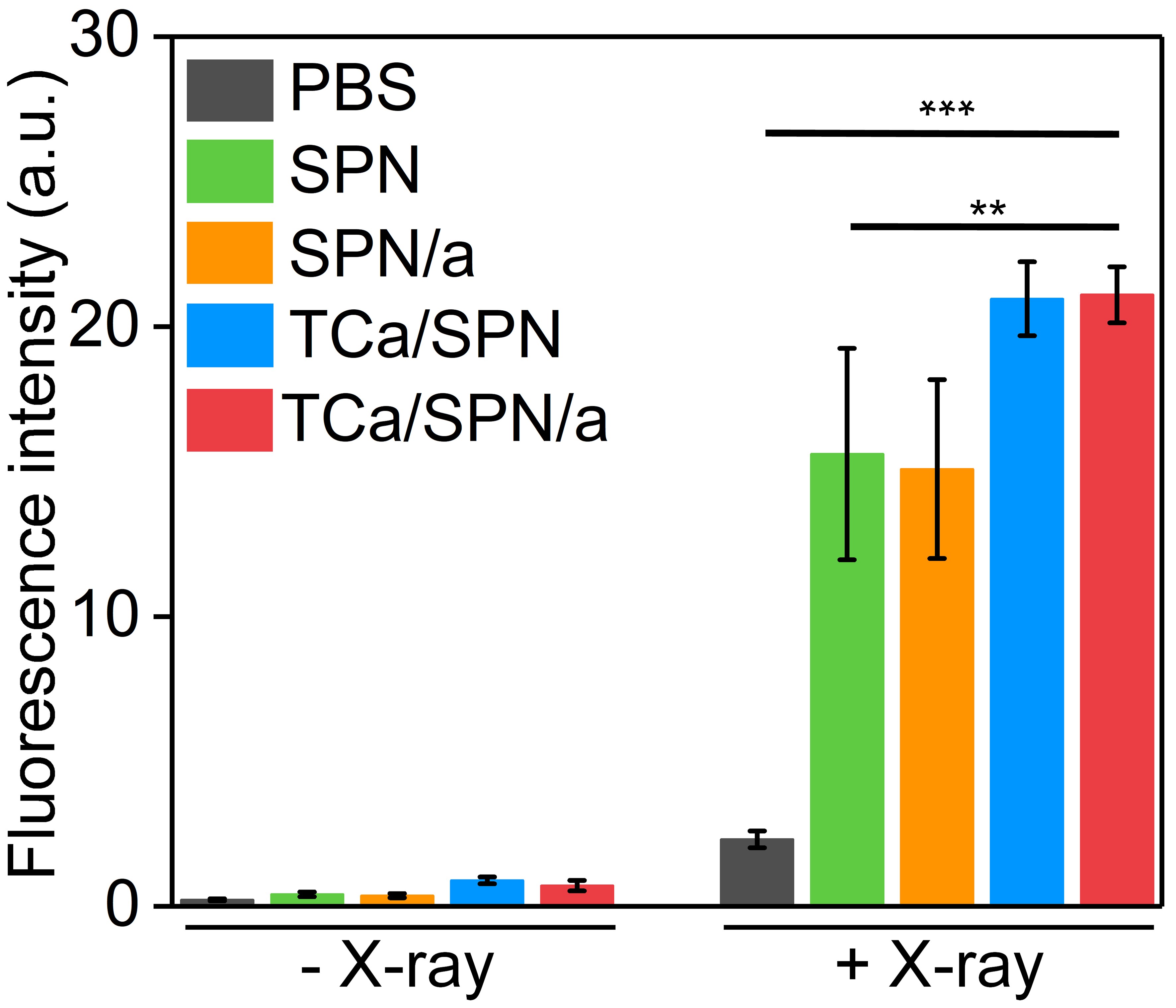


**Figure S12.** Quantitative analysis of CRT fluorescence intensity at tumor sites (n = 5). Data are presented with mean ± SD, (**) p < 0.01, (***) p < 0.001, unpaired two-tailed Student’s t tests.


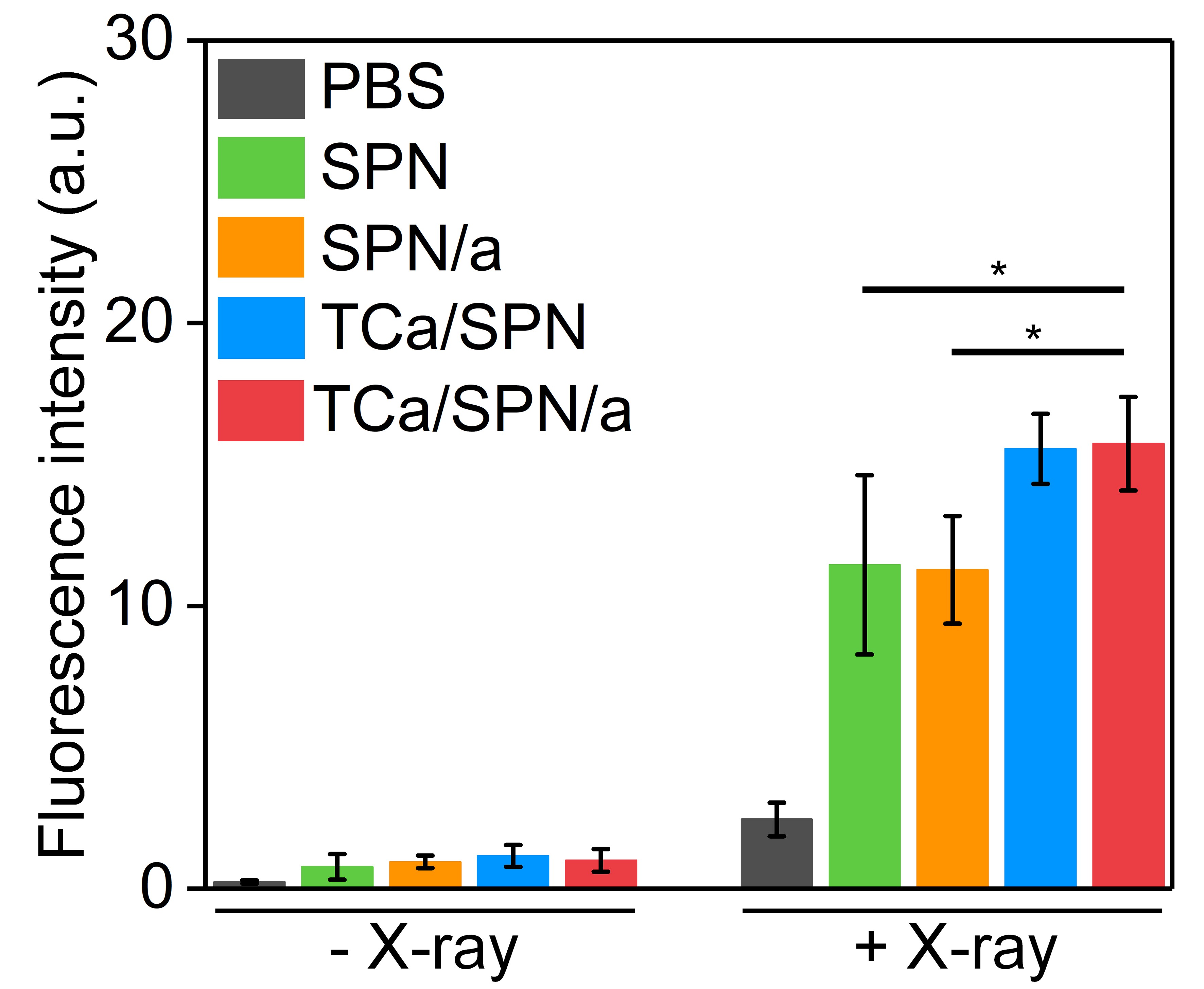


**Figure S13.** The fluorescence intensity of HMGB1 staining signals of tumor tissues (n = 5). Data are presented with mean ± SD, (*) p < 0.05, unpaired two-tailed Student’s t tests.


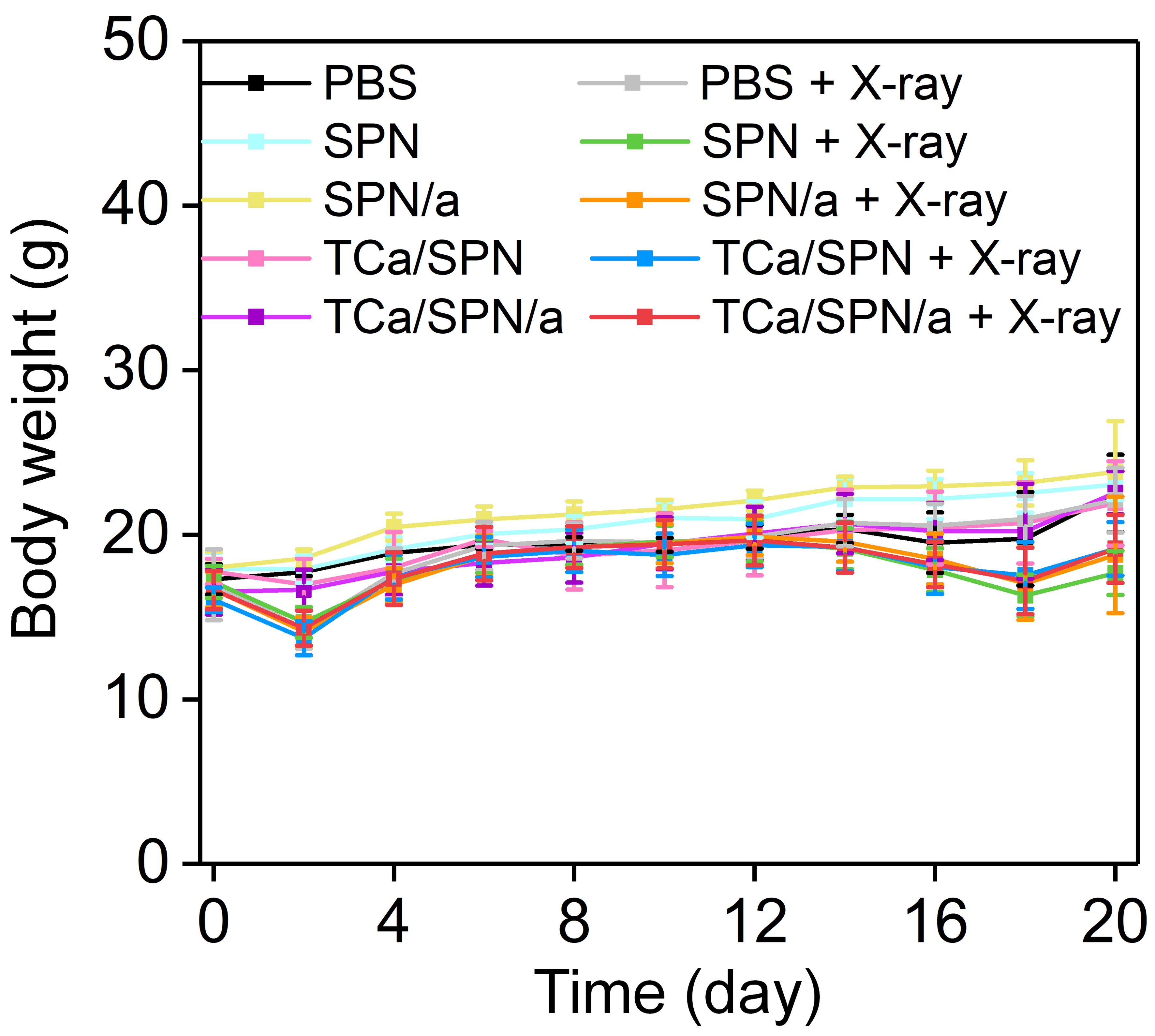


**Figure S14.** The body weight curves of mice in SPN-, SPN/a-, TCa/SPN- and TCa/SPN/a-treated group with or without X-ray irradiation (n = 5). Data are presented with mean ± SD.

**
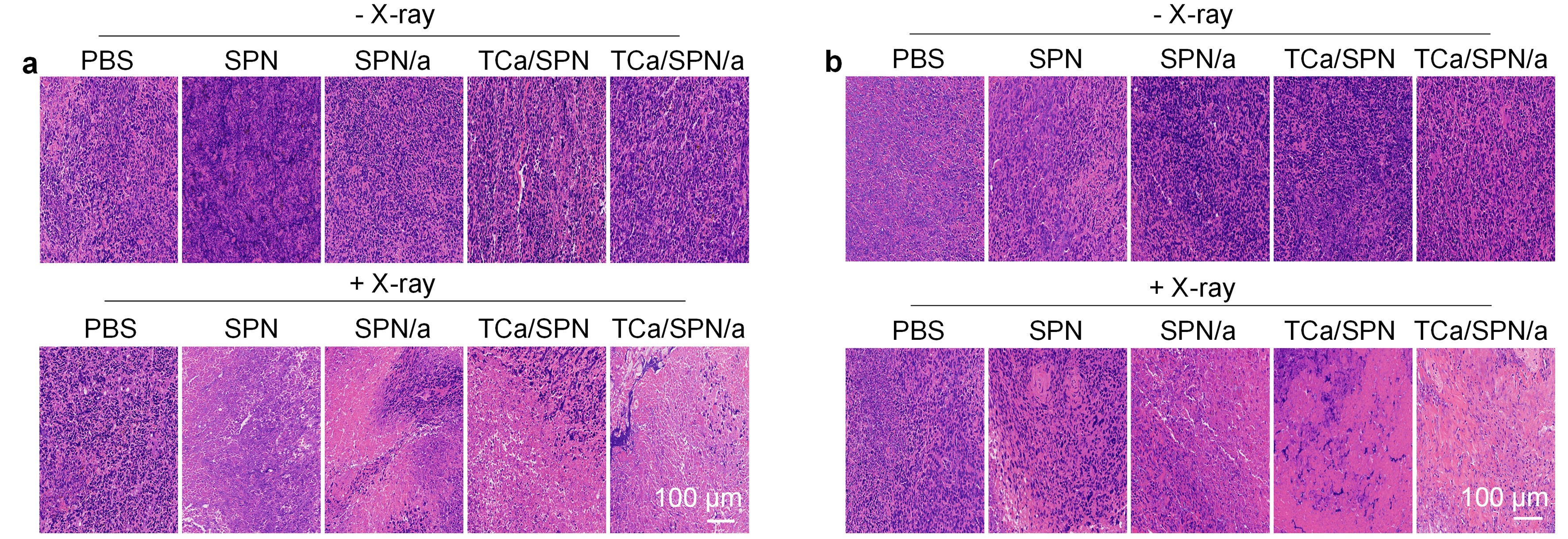
**

**Figure S15.** H&E staining images of (a) primary tumors and (b) distant tumors in SPN-, SPN/a-, TCa/SPN- and TCa/SPN/a-treated mice with or without X-ray irradiation.

**
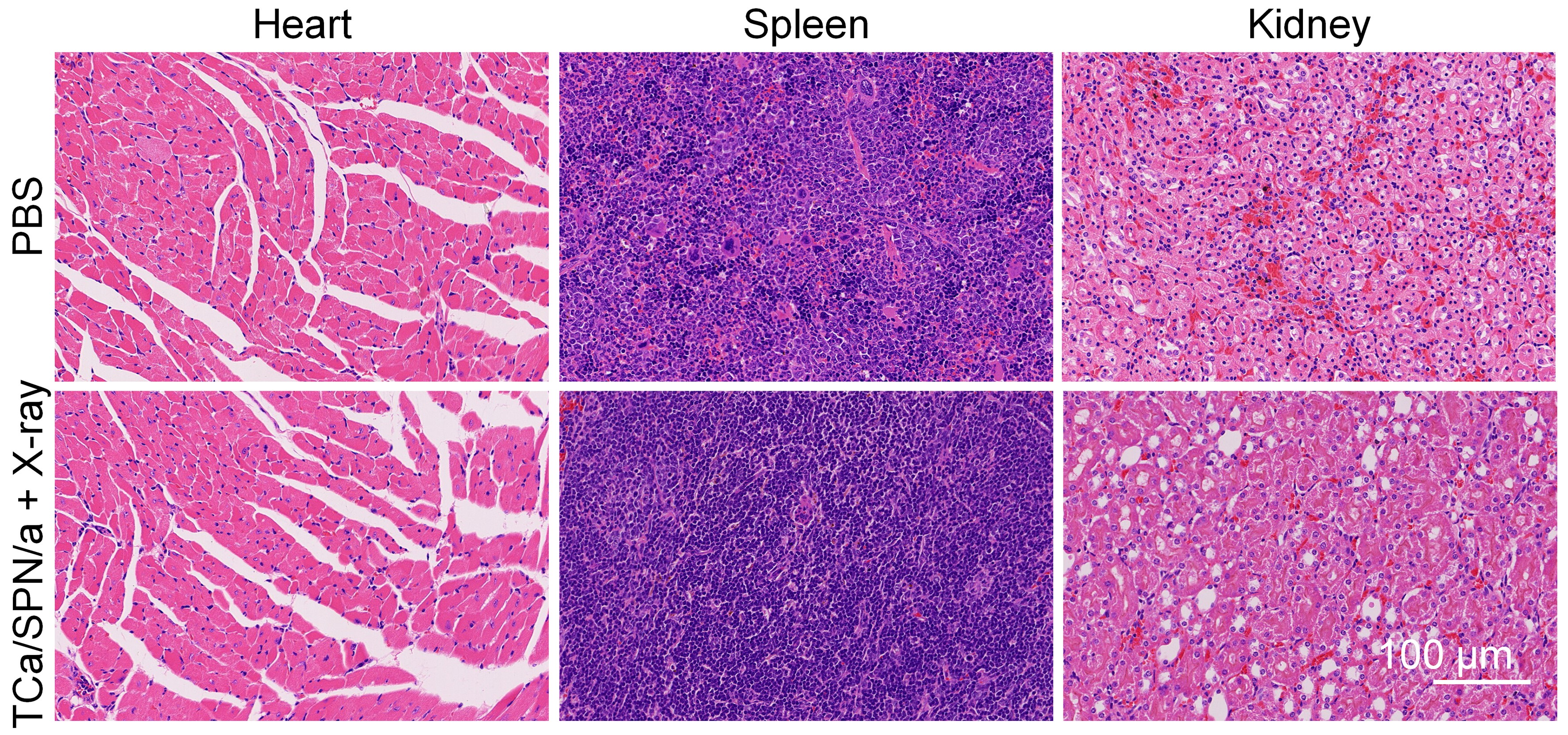
**

**Figure S16.** The H&E staining images of heart, spleen and kidney in different treatment groups.

**
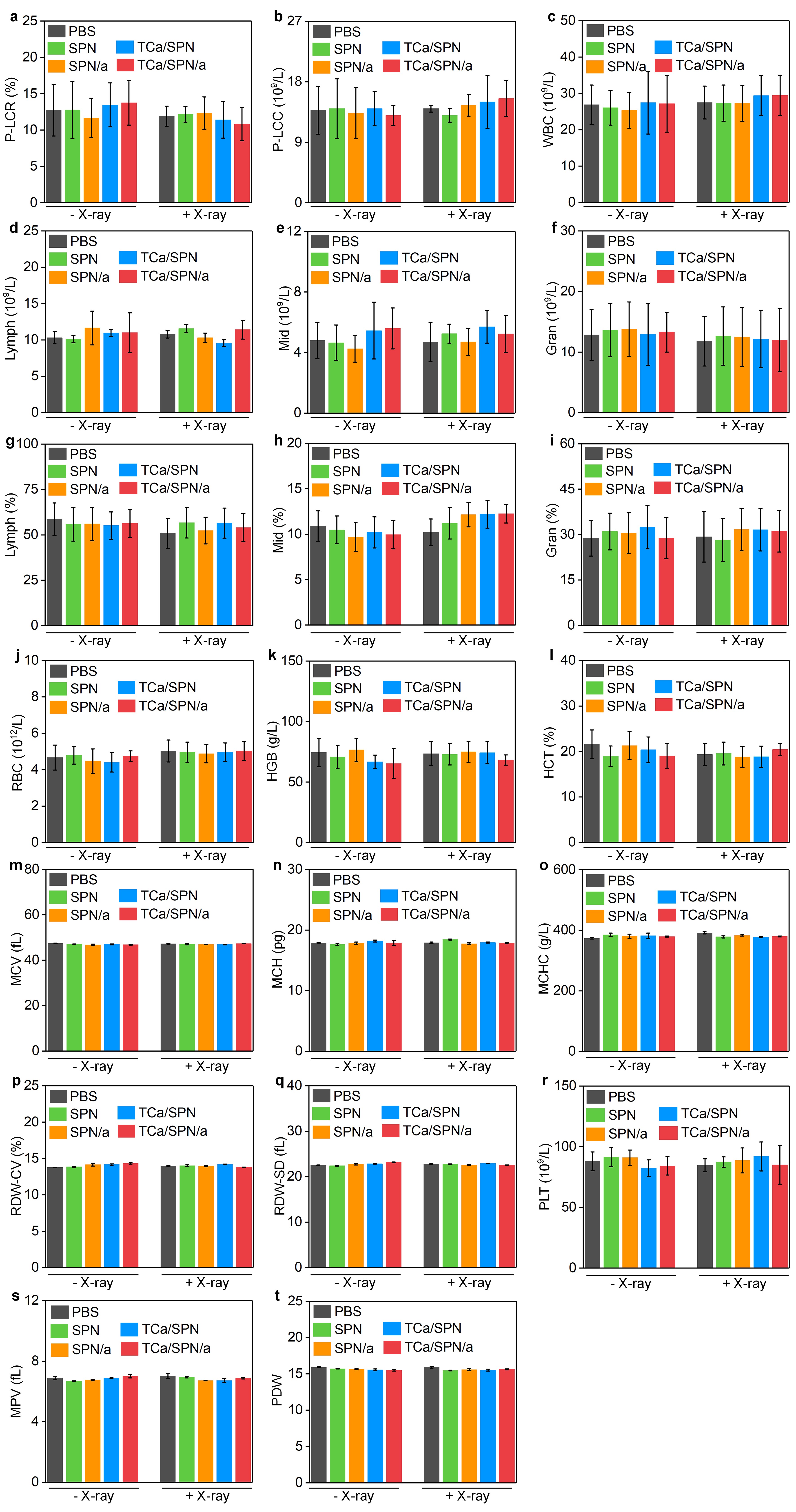
**

**Figure S17.** Assessment of (a) platelet-larger cell ratio (P-LCR), (b) platelet large cell count (P-LCC), (c) white blood cells (WBC), (d) lymph, (e) intermediate cell (Mid), (f) granulocyte (Gran), (g) lymph (%), (h) intermediate cell (Mid) (%), (i) Gran (%), (j) red blood cells (RBC), (k) hemoglobin (HGB), (l) packed cell volume (HCT), (m) mean corpuscular volume (MCV), (n) mean corpuscular hemoglobin (MCH), (o) mean corpuscular hemoglobin concentration (MCHC), (p) red blood cell distribution width-coefficient of variation (RDWCV), (q) red blood cell distribution width-standard deviation (RDWSD), (r) platelet (PLT), (s) mean platelet volume (MPV) and (t) platelet volume distribution width (PDW) in blood samples of treated mice (n = 3). Data are presented with mean ± SD.

**
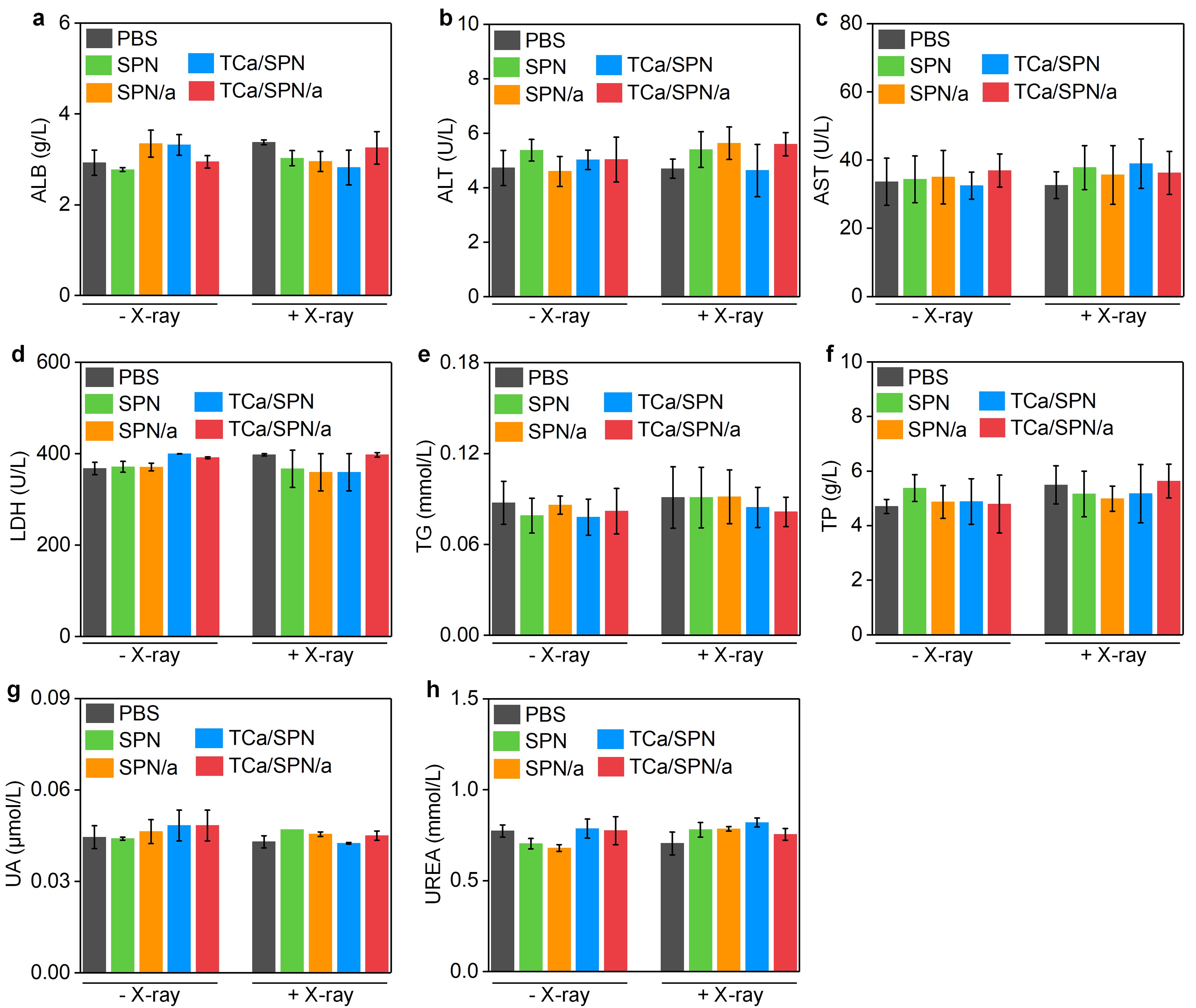
**

**Figure S18.** Detection of (a) albumin (ALB), (b) alanine aminotransferase (ALT), (c) Aspartate aminotransferase (AST), (d) lactate dehydrogenase (LDH), (e) triglyceride (TG), (f) total protein (TP), (g) urine acid (UA) and (h) UREA in various groups of mice (n = 3). Data are presented with mean ± SD.

**
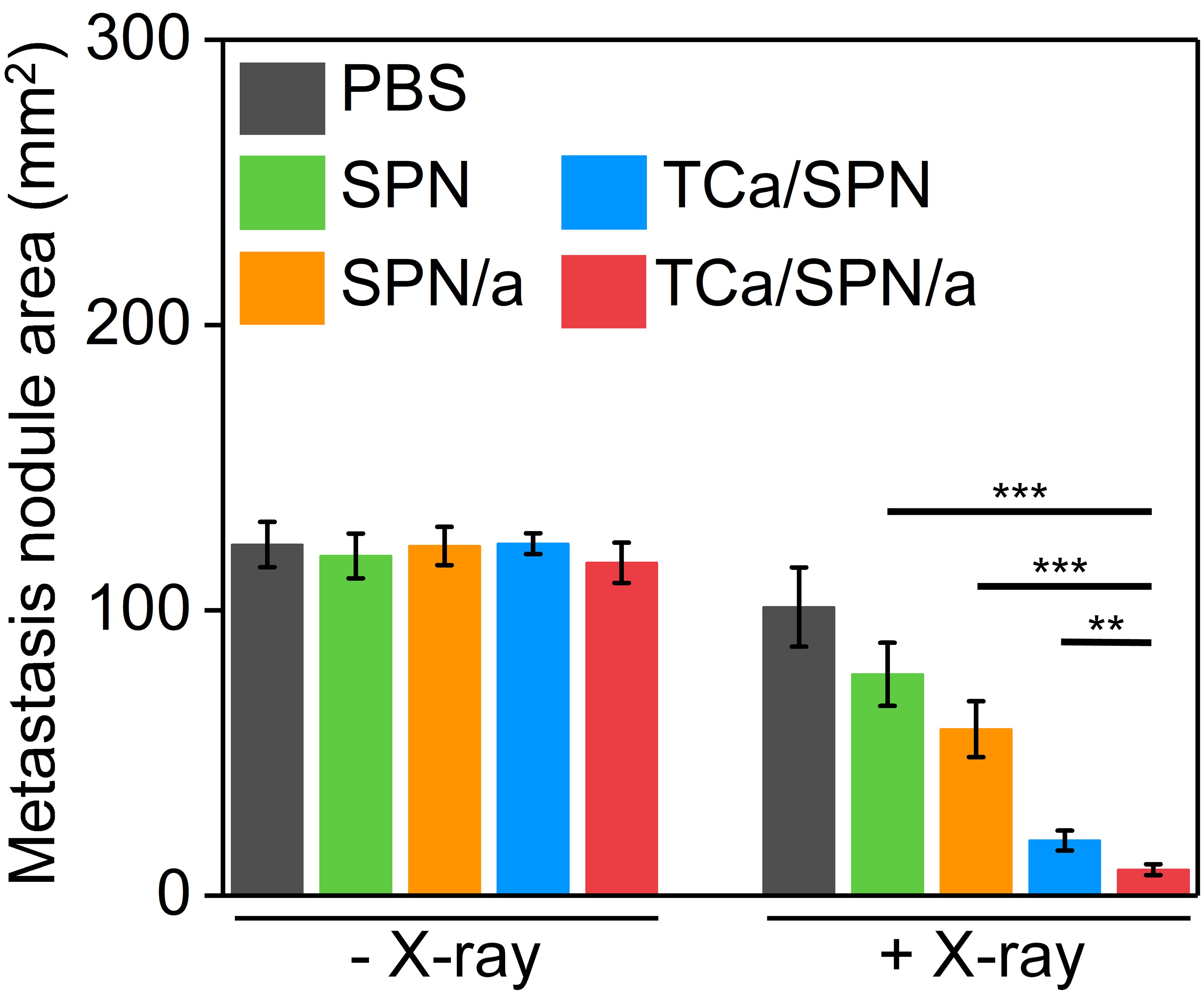
**

**Figure S19.** Quantitative analysis of metastatic area in lung tissues of various treated mice (n = 5). Data are presented with mean ± SD, (**) p < 0.01, (***) p < 0.001, unpaired two-tailed Student’s t tests.


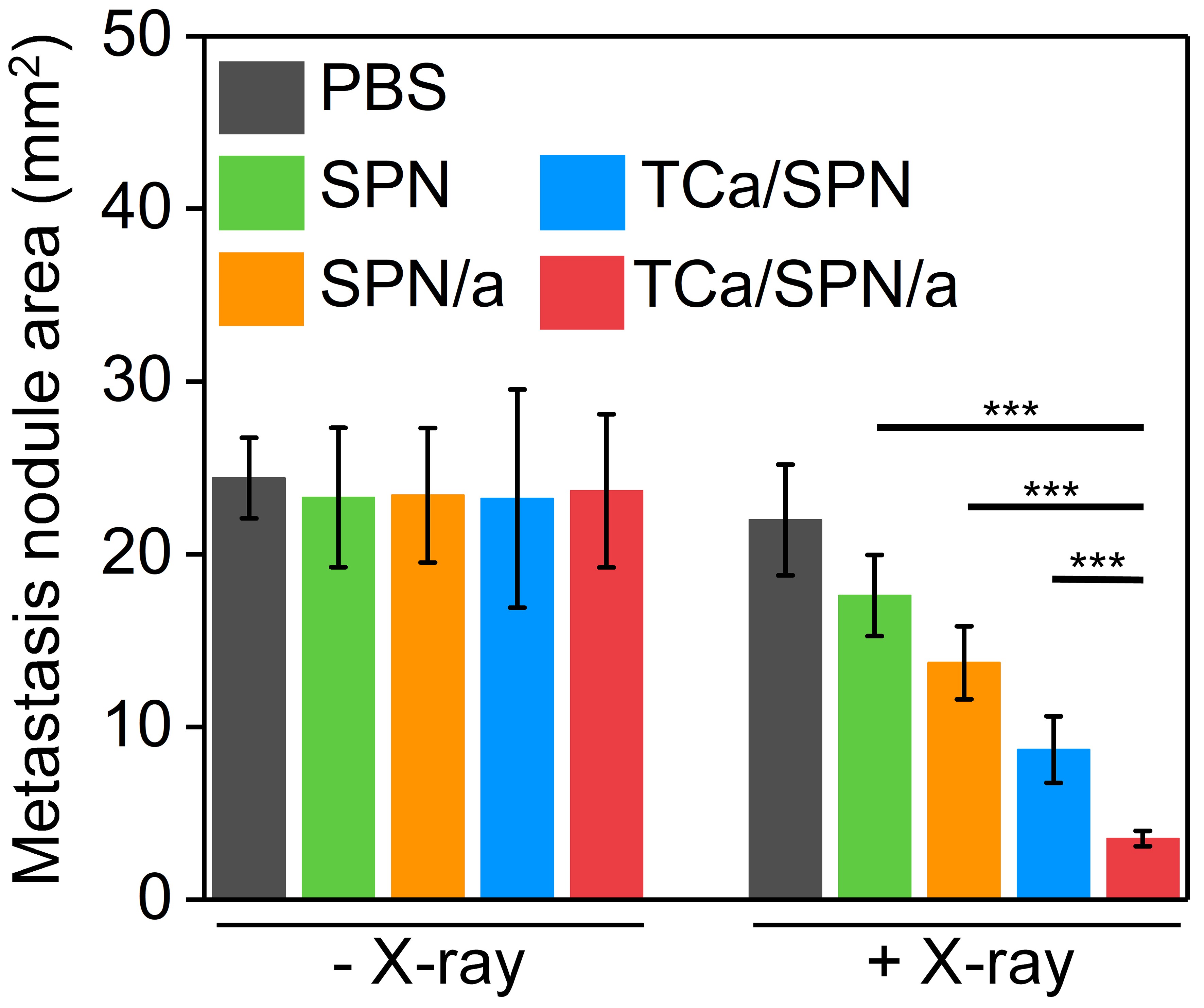


**Figure S20.** Quantitative analysis of metastatic area in liver tissues of various treated mice (n = 5). Data are presented with mean ± SD, (***) p < 0.001, unpaired two-tailed Student’s t tests.


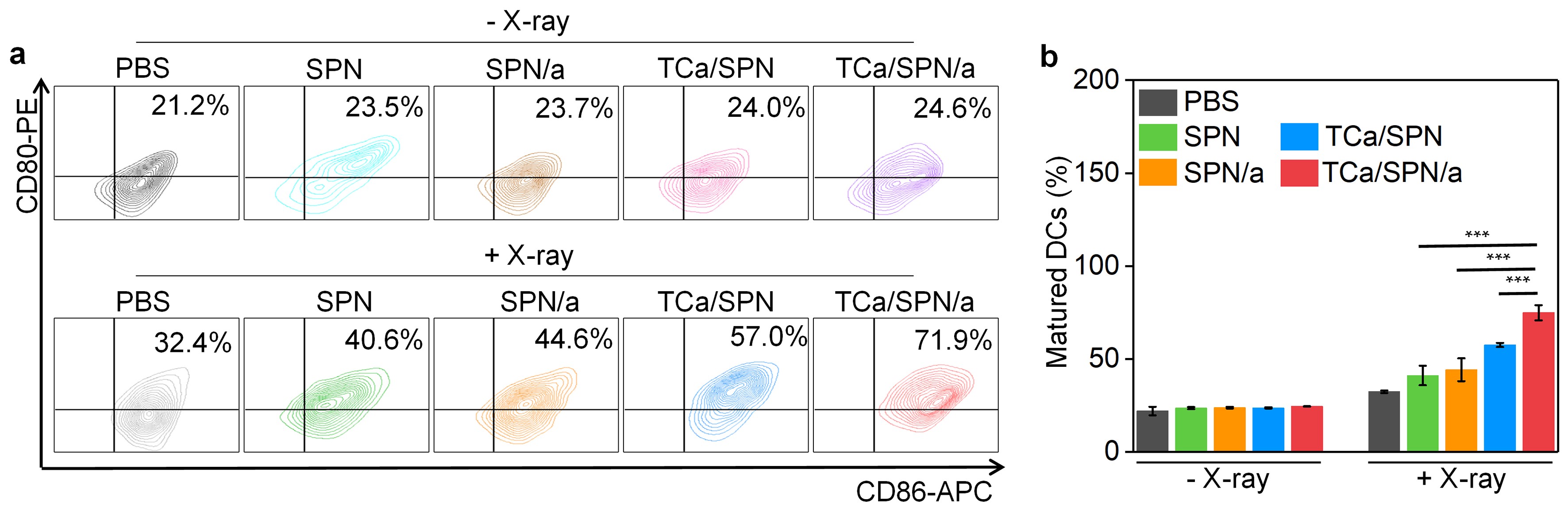


**Figure S21.** (a) Flow cytometer plots of matured DCs in various groups. (b) Quantitative analysis of the percentages of matured DCs (n = 5). Data are presented with mean ± SD, (***) p < 0.001, unpaired two-tailed Student’s t tests.

# 2. Experimental Section

*Materials:* cGAMP was purchased from Selleckchem Inc. (Houston, TX, USA). Triphenylphosphonium (TPP), tetrahydrofuran (THF), trichloromethane acetonitrile and calcium chloride (CaCl_2_) were purchased from Sinopharm (Shanghai, China). Poly[2,7-(9,9-dioctylfluorene)-alt-4,7bis(thiophene-2-yl) benzo-2,1,3-thiadiazole] (PFODBT) and bovine serum albumin (BSA) were gained from Sigma-Aldrich (USA). Hydrogenated soya phosphatidylcholinesn (HSPC) was obtained from Avanti Polar Lipids (Alabaster, USA). 1,2-Distearoyl-sn-glycero-3-phosphorylethanolamine (DSPE) was purchased from MedChemExpress (New Jersey, USA). 1,2-Distearoyl-snglycero-3-phosphoethanolamine-N-[methoxy (polyethylene glycol) (DSPE-mPEG) was gained from Corden Pharma Fribourg (Switzerland). 1,2-Distearoyl-snglycero-3-phosphoethanolamine-thioketal-(polyethylene glycol) (DSPE-TK-PEG) was purchased from Qiyue Biology (Xi’an, China). 1-(3-Dimethylaminopropyl)-3-ethylcarbodiimide (EDC) was obtained from Solarbio (Beijing, China). N-Hydroxysuccinimide (NHS) and 2’,7’-dichlorodihydrofluorescein diacetate (H_2_DCFH-DA) were purchased from Aladdin Industrial Corporation (USA). Singlet oxygen sensor green (SOSG) was obtained from Thermo Fisher Science Co., Ltd. (Invitrogen, USA). Mouse high mobility group protein B1 (HMGB1) ELISA kit was purchased from Colorful Gene Biological Technology Co., Ltd (Wuhan, China). Cell counting kit-8 (CCK-8) was obtained from Meilun Biotechnology Co., Ltd (Dalian, China). Adenosine triphosphate (ATP) detection kit, mitochondrial membrane potential assay kit, DNA damage detection kit and nuclear fluorescence dye 4’,6-diamidino-2-phenylindole (DAPI) were purchased from Beyotime Biotechnology Co., Ltd. (Shanghai, China). Anti-calreticulin (CRT) antibody was obtained from Abcam Inc. (Cambridge, MA, USA). Alexa Fluor 488-labeled goat anti-rabbit IgG (H + L) was gained from Beyotime Biotechnology Co., Ltd. (Shanghai, China). All antibodies for flow cytometry analysis were acquired from Biolegend (San Diego, CA, USA) and BD Biosciences (San Jose, CA, USA). Dulbecco’s modified Eagle’s medium (DMEM), penicillin-streptomycin and trypsin were acquired from Servicebio (Wuhan, China).

*Characterization:* ^1^H NMR spectra were obtained using a Bruker Biospin AVANCE400 spectrometer. Ca content was detected using inductively coupled plasma-optical emission spectrometry (ICP-OES, USA). The morphology analysis was performed using a transmission electron microscope (TEM) (JEM-2100, JEOL, Japan). The size, polydispersity index (PDI) and zeta potentials were measured by Malvern Zetasizer Nano ZS90 (England). Absorption spectra were measured on ultraviolet-visible absorption spectrometry (TU-1810, Persee, Beijing, China). The fluorescence spectrophotometer (RF-6000, SHIMADZU, Japan) was utilized to recorded the fluorescence spectra. Analysis of cGAMP release was performed using high-performance liquid chromatography (HPLC) (SHIMADZU, Japan). The fluorescence images were obtained using confocal laser scanning microscope (CLSM, LSM700, Carl Zeiss, Germany) and inverted fluorescence microscope (DMi8, Leica, Germany). Fluorescence images of living animals was recorded by IVIS Lumina Series III system (PerkinElmer, USA). X-ray irradiation was performed using MultiRad 225 X-ray device (Tucson, Arizona, USA). Blood biochemistry analysis was performed using automatic biochemical analyzer (Chemray 800, China). Blood routine examination was performed using automatic blood cell analyzer (BC-30, China). The immune cells were analyzed using flow cytometry (BD FACS Melody, USA).

*Cell Culture:* 4T1 breast cancer cells line was obtained from American Type Culture Collection. 4T1 cancer cells were cultured in DMEM medium contained 10% FBS, 100 U/mL penicillin and 0.1 mg/mL streptomycin.

​​​​​​​​​​​*Cytotoxicity Analysis:* 4T1 cells were cultured in 96-well plate (1 × 10^4^ cells/well) and cultured in DMEM containing SPN, SPN/a, TCa/SPN and TCa/SPN/a (0, 1.3, 2.5, 5,10, 20, 50 and 100 μg/mL) for 24 h. CCK-8 kit was utilized to evaluate the viability of these treated cells.

*In Vitro Therapeutic Effect Analysis:* 4T1 cancer cells were seeded in 96-well plate and divided into 10 groups randomly to investigate the treatment effect. In brief, 4T1 cancer cells were incubated with PBS, SPN, SPN/a, TCa/SPN or TCa/SPN/a (20 μg/mL) for 12 h and then exposed under 6 Gy of X-ray irradiation. These treated cells were cultured for another 12 h and the cell viability was measured using CCK-8 kit.

*Measurement of ROS Generation In Vitro:* 4T1 cancer cells were cultured in 24-well plate (2 × 10^5^ cells/well) and incubated with PBS, SPN, SPN/a, TCa/SPN or TCa/SPN/a (20 μg/mL) for 8 h. H_2_DCFH-DA probe (10 µM/well) was added in each well and the cells were incubated for 30 min. The cells were then treated with X-ray irradiation (6 Gy). Finally, the cells were observed using inverted fluorescence microscope to evaluate generation of ROS inside cells.

*Cell Uptake Efficacy Analysis:* 4T1 cells were seeded in cell round coverslip and co-incubated with PBS, SPN, SPN/a, TCa/SPN or TCa/SPN/a (20 μg/mL) for 8 h. The cells were washed by 1 × PBS to remove excess nanoparticles and Hoechst (1:1000 dilute) was utilized to stain the nucleus for 30 min. Finally, fluorescence images of the cells were obtained using scanning confocal microscope. In addition, the treated 4T1 cells were digested after DAPI staining (1:500 dilute) for 30 min and then filtered through a 70 μm filter, and the uptake efficacy was detected by flow cytometry.

*DNA Damage Evaluation:* 4T1 cells were cultured on cell round coverslip in 6-well plate and co-incubated with PBS, SPN, SPN/a, TCa/SPN or TCa/SPN/a (20 μg/mL) for 8 h. Then, the cells were exposed to X-ray irradiation (6 Gy), washed with 1 × PBS for at least 3 times, and fixed with 4% formaldehyde for 30 min. After that, γ-H2AX murine monoclonal antibody was used to stain the cells at room temperature for 1 h. Subsequently, the cells were stained with Alexa Fluor 488-labeled goat anti-rabbit IgG (H + L) for 1 h and DAPI for 30 min, followed by washing with 1 × PBS. The fluorescence signals of cells were observed to evaluate DNA damage.

*Detection of CRT Exposure and HMGB1 Release:* 4T1 cells were incubated with PBS, SPN, SPN/a, TCa/SPN and TCa/SPN/a (20 μg/mL) for 8 h and then treated with X-ray irradiation (6 Gy). After treatments, the cells were incubated for another 24 h and immersed in 1% BSA solution containing ​anti-CRT antibody (1:1000 dilute) at 37 ℃ for 1 h. Then the cells were incubated with Alexa Fluor 488-labeled Goat Anti-Rabbit IgG (H + L). Images of CRT immunofluorescence staining were obtained by CLSM and fluorescence intensity of cells was quantified by image J software. In addition, the treated cells were used to collect supernatant through centrifugation (2000 rpm, 4 ℃). The HMGB1 levels of the supernatant were tested according to the instructions of HMGB1 kit. The HMGB1 contents were calculated according to the standard curve.

*Analysis of Extracellular ATP Levels:* After incubation of 4T1 cells with PBS, SPN, SPN/a, TCa/SPN and TCa/SPN/a (20 μg/mL) and X-ray irradiation (6 Gy), the cells were used to determine the levels of ATP using assay kit.

*Mitochondrial Membrane Potential Change Analysis:* 4T1 cells were incubated with PBS, SPN, SPN/a, TCa/SPN and TCa/SPN/a (20 μg/mL) for 8 h, followed by 6 Gy of X-ray irradiation. The treated cells were incubated with JC-1 probe at 37 ºC for 20 min, and the fluorescence signals of cells were observed using CLSM.
